# Modelling T cell proliferation: Dynamics heterogeneity depending on cell differentiation, age, and genetic background

**DOI:** 10.1371/journal.pcbi.1005417

**Published:** 2017-03-13

**Authors:** Julien Vibert, Véronique Thomas-Vaslin

**Affiliations:** Sorbonne Universités, UPMC Univ Paris 06, INSERM, Immunology-Immunopathology-Immunotherapy (I3) UMRS959; Paris, France; Imperial College London, UNITED KINGDOM

## Abstract

Cell proliferation is the common characteristic of all biological systems. The immune system insures the maintenance of body integrity on the basis of a continuous production of diversified T lymphocytes in the thymus. This involves processes of proliferation, differentiation, selection, death and migration of lymphocytes to peripheral tissues, where proliferation also occurs upon antigen recognition. Quantification of cell proliferation dynamics requires specific experimental methods and mathematical modelling. Here, we assess the impact of genetics and aging on the immune system by investigating the dynamics of proliferation of T lymphocytes across their differentiation through thymus and spleen in mice. Our investigation is based on single-cell multicolour flow cytometry analysis revealing the active incorporation of a thymidine analogue during S phase after pulse-chase-pulse experiments *in vivo*, versus cell DNA content. A generic mathematical model of state transition simulates through Ordinary Differential Equations (ODEs) the evolution of single cell behaviour during various durations of labelling. It allows us to fit our data, to deduce proliferation rates and estimate cell cycle durations in sub-populations. Our model is simple and flexible and is validated with other durations of pulse/chase experiments. Our results reveal that T cell proliferation is highly heterogeneous but with a specific “signature” that depends upon genetic origins, is specific to cell differentiation stages in thymus and spleen and is altered with age. In conclusion, our model allows us to infer proliferation rates and cell cycle phase durations from complex experimental 5-ethynyl-2'-deoxyuridine (EdU) data, revealing T cell proliferation heterogeneity and specific signatures.

## Author summary

We assess the impact of genetics and aging on immune system dynamics by investigating the dynamics of proliferation of T lymphocytes across their differentiation through thymus and spleen in mice. Understanding cell proliferation dynamics requires specific experimental methods and mathematical modelling. Our investigation is based upon single-cell multicolour flow cytometry analysis thereby revealing the active incorporation in DNA of a thymidine analogue during S phase after pulse-chase experiments in vivo, versus cell DNA content. A generic mathematical model that simulates the evolution of single cell behaviour during the experiment allows us to fit our data, to deduce proliferation rates and mean cell cycle phase durations in sub-populations. This reveals that T cell proliferation is constrained by genetic influences, declines with age, and is specific to cell differentiation stage, revealing a specific “signature” of cell proliferation. Our model is simple and flexible and can be used with other pulse/chase experiments.

## Introduction

Cell division is a characteristic of biological systems, with variability of rates of proliferation and interphase duration according to the type of organism, organ, and period of life. In the immune system, cell proliferation is essential for the high turnover of diversified lymphocytes that insures dynamic cognitive function and maintenance of body integrity. The production of T cells occurs through processes of proliferation, differentiation, migration, selection and death in the thymus to finally export less than 6% of cells to peripheral tissues as naïve T cells [1]. T cells circulate through the whole organism and interact with environmental and body antigens to proliferate and differentiate into effector/memory T cells upon antigenic recognition. Quantification and interpretation of lymphocyte dynamics remains a challenge in systems immunology [2, 3]. Studies on T cell dynamics at various ages have questioned the mechanisms of thymic involution [4] beginning at puberty, followed by immuno-senescence and inflammation with aging [5], and physiological accumulation of effector/memory peripheral T cell clones, particularly of CD8 phenotype, in both aged humans [6] and mice [7]. Although the number of T cells decreases with aging, promiscuous CD4 T cells accumulate [8]. Thymic hypoplasia or thymectomy in young individuals also affects the repertoire of peripheral T cells and immunocompetence [9]. Thus, thymic production is primordial so as to maintain a homeostatic and dynamic equilibrium in terms of cell populations and repertoires, thereby allowing an effective and adaptive immune surveillance. Sequential T cell development and CD4/CD8 lineage decision models were previously described [10–13]. However, there remain open questions concerning the rates of thymocyte production that result from various processes, such as differentiation, proliferation, selection, and death of T cells, and that influence the homeostasis and life-span of naïve and effector/memory T cells in secondary lymphoid organs. Several theoretical approaches and experimental immunological protocols are available to investigate lymphocyte dynamics [3] and to model them [14]. A number of studies have tried [15–17], often with the help of modelling [1, 18–21], to quantify thymocyte production, thymic output, and the processes involved in maintenance of a T lymphocyte dynamic equilibrium in the periphery in humans or mice [1, 22–26]. It was suggested that the naïve T cell pool is maintained by peripheral division in humans, at variance to thymic output in mice, and with significant differences in lifespan of CD8 peripheral T cells in young and old C57BL/6 mice [27]. Some findings also suggested different genetic regulations of thymic output according to strains of mice [28]. The flexibility of T cell behaviour has already been approached, in particular for peripheral cells [29], but references concerning quantitative proliferation are still lacking.

Altogether, these studies were performed with various experimental methods, on various species, strains, ages, and cell populations. Consequently, no clear consensus has been reached concerning the influence of genetics and age on T cell dynamics. We have previously observed that both the genetic background and aging affect T cell dynamics and repertoires [30]. Indeed, C57BL/6 and FVB mice differ in their T cell composition at steady state but also in their kinetics of T cell reconstitution after a transient immunosuppression. In FVB mice, there is an acceleration of thymic involution and of the immunological aging quantified by alterations in the homeostatic control of lymphocyte numbers and repertoire diversity in the periphery [30].

To determine whether or not the differences in the dynamic behaviour of T cells previously observed between the FVB and B6 mice strains are related to the proliferation capacities of T cells during their differentiation, we assayed cell proliferation *in vivo* by active DNA labelling of cells in S phase. We designed an original *in vivo* cell labelling experiment with 5-ethynyl-2'-deoxyuridine (EdU) during a pulse-chase-pulse experiment, followed by multi-parameter flow cytometry analysis, on the basis of earlier methods that measured bivariate amounts of incorporated label versus DNA content in single cells [15, 31]. To our knowledge, there is currently no model able to analyse the bi-dimensional dot plot flow cytometry data, in order to infer dynamic parameters of cell cycle phases after complex pulse-chase experiments. Thus, to interpret the complex cell dynamics during the experiment, we designed a generic mathematical model that describes the dynamics of cells that transit from G0/G1 to S and then to G2/M phase of the cell cycle according to variable periods of pulse-chase. Fitting of the model to the experimental bi-dimensional flow cytometry data allowed us to infer rates of proliferation, and to give an estimate of mean duration times of cell cycle phases. Our mathematical model is also able to fit data from other pulse/chase experiment protocols and by extension can potentially be used to investigate cell cycle kinetics in any cell type.

This study provides a detailed proliferation assessment and cell quantification in subpopulations of T cells, along their differentiation from thymus to spleen, and through aging. For the first time, we show that genetic origin and age drive a particular "signature" of proliferation and cycle phase durations, according to T cell differentiation stage and T cell lineage. FVB mice have significantly lower rates of T cell proliferation than C57BL/6 mice in both thymus and spleen. Proliferation decreases with age in both strains. These results are discussed in the perspective of T cell dynamics and proliferation of T cells, where aging and genetic peculiarities could be of importance.

## Results

### Assessment of in vivo cell proliferation by active DNA labelling

**Fig 1:**
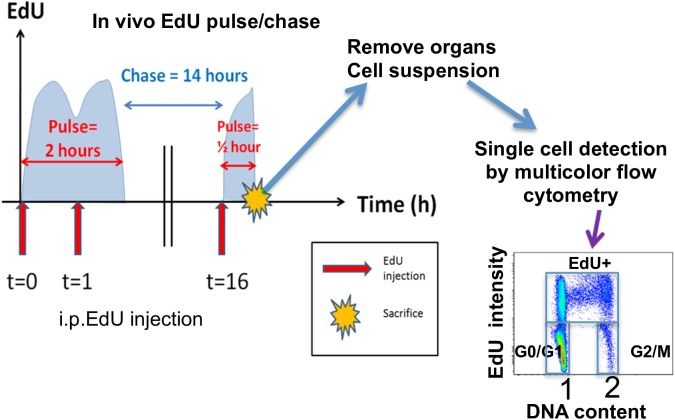
EdU pulse chase experiment for quantification of cell proliferation. Mice were injected intra-peritonealy with EdU at 0, 1, and 16 hours equivalent to a "pulse-chase-pulse" experiment. Thirty minutes after the last pulse, the mice were sacrificed, lymphoid organs removed, and cell suspensions were analysed by single-cell multicolour flow cytometry. The bi-dimensional dot plot reveals the intensity of EdU label (log scale) and DNA content (linear scale arbitrary unit) in lymphocytes. Manual gating allows identification of unlabelled cells in either G0/G1 (G) or G2/M (M) that have not transited through S phase during pulse, and EdU labelled cells (G'+S'+M') that have transited through S phase.

To determine proliferation properties of cell populations, active labelling of cells during S phase of the cell cycle was done with EdU, a thymidine analogue, during pulse/chase periods. In our specific case, two pulse periods were separated by a fourteen-hour chase interval (Fig 1). Since the *in vivo* half-life of EdU is about one hour [32], this was equivalent to a pulse-chase-pulse experiment comprising an initial two-hour pulse followed by a fourteen-hour chase and then a second thirty-minute pulse. This protocol was chosen because it allows the labelling of cell populations with low proliferation rates, which would not be detected with only one EdU injection. Multicolour flow cytometry then allowed the identification of the cell cycle phases and the quantitative active EdU labelling status, using bi-dimensional EdU label/DNA content dot plots that represent single cell analysis. According to DNA content, cells can be classified in G0/G1 phase (DNA content equal to 1), G2/M phase (DNA content equal to 2), or S phase (DNA content between 1 and 2). According to the intensity of EdU label, one can distinguish between labelled cells that have progressed through S phase during the pulse, and non-labelled cells. Thus, the dot plots represent the end snapshot of the cell population evolution through periods of pulse/chase. Three groups of cells can be delimited on our dot plots (Fig 1): G0/G1 unlabelled, G2/M unlabelled, and EdU labelled cells. Since the experiment ends with a pulse phase, there are no remaining unlabelled cells in S phase.

### Differential cell proliferation in T cells according to differentiation stage, age and strain

**Fig 2:**
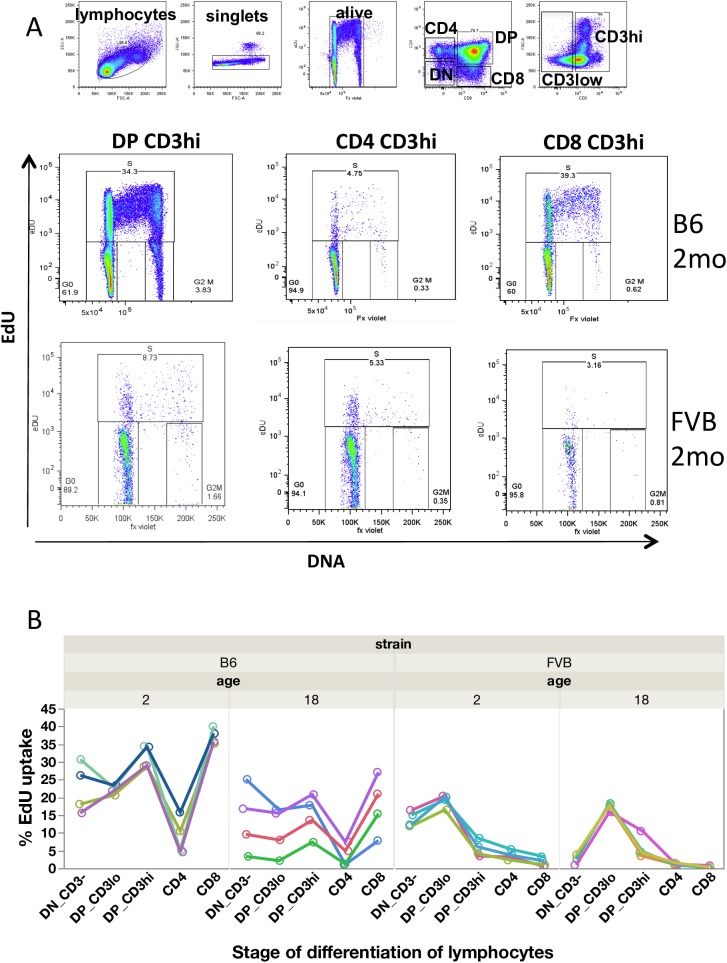
Thymocyte proliferation in mice according to cell differentiation stage, age and genetic origin. Cell proliferation was assessed in C57BL/6 and FVB mice of 2 or 18 months as in Fig 1. (A) Multi-colour flow cytometry and hierarchical gating in thymocytes showing EdU vs DNA content dot plot in 2-month-old mice. (B) Percentage of EdU^+^ labelled cells according to differentiation stages through time: from the most immature CD3^-^CD4^-^CD8^-^ triple negative cells (TN), CD3^lo^CD4^+^CD8^+^ double positive cells (DP CD3^lo^), CD3^hi^CD4^+^CD8^+^ double positive cells (DP CD3^hi^) to single positive mature CD4^+^CD3^+^ or CD8^+^CD3^+^stage. Each curve represents the %EdU uptake for one of the 4 mice per group.

Our purpose was to assess the variation of T cell dynamics and heterogeneity in individuals, in time and location, by quantification of T cell proliferation in two different strains (FVB and B6), through aging (at 2 and 18 months) and during differentiation in lymphoid organs (from thymus to spleen). As exemplified in Fig 2A, in the thymus, the percentage of EdU labelled cells varies significantly according to strains of mice and the stage of thymocyte differentiation. Thus, comparing the FACS profiles between typical 2-month-old FVB and B6 mice, one can observe that the proportion of EdU^+^ cells is much lower in FVB mice, showing lower proliferation capacities in this strain. A given group of mice depicts a particular signature of proliferation according to strain and age (Fig 2B) revealing the heterogeneity of proliferation across the stages of differentiation. The percentages of labelled cells after 16 hours are given in S2 Table. With aging, at 18 months, which corresponds to middle life in mice, the proliferation of thymocytes in B6 is reduced by about 1.5 fold in all populations. In 18-month-old FVB mice, the proliferative capacities are even more reduced in immature CD3^-^CD4^-^CD8^-^ TN cells and thereafter are quite comparable to the altered proliferation capacities already detected in young FVB mice. In conclusion, T-cell dynamics is very heterogeneous, depending on the differentiation stages as detected by the variation in the EdU uptake. Cell proliferation is altered with age, and genetic origin influences the proliferation capacities, which are depressed in FVB mice. Thus, a typical biological signature of thymocytes is revealed, allowing one to distinguish strains of mice and ages, although precise quantification of dynamic parameters is required.

### Model of labelling experiment and fit to experimental data

**Fig 3:**
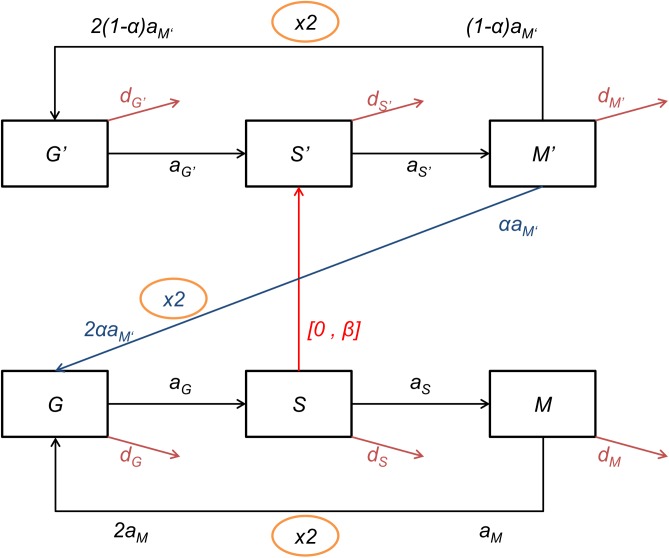
State transition model of cell cycle and EdU labeling. According to the FACS dot plot, the evolution of cells through the cell cycle and EdU labelling can be represented by a transition diagram. Unlabelled cells can transit successively in the three phases of the cell cycle: G0/G1 (G), S (S), and G2/M (M). Terms in a_X_ correspond to rates of entry into the next phase of cells in X phase (X being either G, S, or M). The exit of cells, either due to death, differentiation, or migration is represented by terms in d_X_. During the pulse-phase the cells in S phase incorporate EdU (red arrow) and enter into S’ with rate β and continue the cycle to M’ and G’. During the chase phase, in the absence of EdU, the labelled cells lose labelling upon several cell divisions (blue arrow) (de-labelling) with rate α. Unlabelled cells can enter into S phase but remain unlabelled.

Our aim was to quantitatively estimate proliferation parameters and mean life-span of cells (here restricted to inter-mitotic time), according to age and strain, in a standard way independent of pulse-chase experiment duration, and to express results as a proliferation rate/day. Thus we designed a mathematical model to interpret and fit the data obtained from the experimental cytometry bi-dimensional dot plots. To model system dynamics and the variation of “stocks” of cells as a function of time, equivalent representations can be proposed: the hydraulic metaphor, stock-flow/transition diagrams, integral equations, and differential equations [33]. We chose to explain our model with a state-transition diagram (Fig 3), a visual language that is accessible to biologists and provides an equivalence to a system of ODEs [34, 35]. A state-transition diagram allows representation of the parallel processes occurring in cells and during the experiment. Through time, the cells can progress across three stages, G0/G1, S, G2/M or exit the population (death, differentiation, migration). During the alternative phases of pulse and chase of EdU, the cells can exist in one of two stages, either EdU unlabelled (Edu^-^) or EdU labelled (EdU^+^). Thus, by combination, the cells can progress in six different stages (Table 1) represented by six differential equations (Eq 1). Moreover, the cells progress through the cycle in the context of EdU bio-availability (pulse or chase). Altogether, the multiplicity of the states (3x2x2) results in a system of twelve equations (see Materials and Methods section and Tables 1 to 3 for the description of the mathematical model).

**Table 1:** Cell populations described in the model (Fig 3) and initial values used in the fit according to our hypotheses.

Population name	Population description	Initial value used for the fit
*G*	Cells in G0 or G1 phase, unlabelled	${\left(G+G\prime \right)}_{ss}=\frac{200a_{M}(a_{S}+d_{S})}{2a_{M}(a_{S}+d_{S})+2a_{M}a_{G}+(a_{G}+d_{G})(a_{S}+d_{S})}$
*S*	Cells in S phase, unlabelled	${\left(S+S\prime \right)}_{ss}=\frac{200a_{M}a_{G}}{2a_{M}(a_{S}+d_{S})+2a_{M}a_{G}+(a_{G}+d_{G})(a_{S}+d_{S})}$
*M*	Cells in G2 or M phase, unlabelled	$({M+M\prime )}_{ss}=\frac{100(a_{G}+d_{G})(a_{S}+d_{S})}{2a_{M}(a_{S}+d_{S})+2a_{M}a_{G}+(a_{G}+d_{G})(a_{S}+d_{S})}$
*G'*	Cells in G0 or G1 phase, labelled	0
*S'*	Cells in S phase, labelled	0
*M'*	Cells in G2 or M phase, labelled	0

**Table 2:** Model parameters and values used for the fit in our experimental conditions.

Parameter name	Parameter description	Value used for the fit
a_G_, a_G'_	Rate of entry into S phase of G1 phase unlabelled, labelled cells	a_G_ = a_G '_ = parameter to fit
a_S_, a_S'_	Rate of entry into G2 phase of S phase unlabelled, labelled cells	a_S_ = a_S '_ = 1/6.5
a_M_, a_M'_	Rate of entry into cell division of M phase unlabelled, labelled cells	a_M_ = a_M '_ = parameter to fit
d_G_, d_G'_	Rate of cell death, differentiation or migration of G0/G1 phase unlabelled, labelled cells	d_G_ = d_G'_ = a_G_
d_S_, d_S'_	Rate of cell death, differentiation or migration of S phase unlabelled, labelled cells	d_S_ = d_S'_ = 0
d_M_, d_M'_	Rate of cell death, differentiation or migration of G2/M phase unlabelled, labelled cells	d_M_ = d_M'_ = 0
α	Proportion (0≤α≤1) of labelled M phase cells shedding label during cell division	α = 0 (no cell loses its label with cell division)
β	Rate of labelling of S phase cells during the pulse phase	β = ∞ (instant labelling by EdU)
p	Proliferation rate	Result of the fit

**Table 3:** Biological and experimental hypotheses, allowing for constraint of parameter values.

Hypothesis number	Hypothesis description	Consequence for parameters
1	EdU labelling does not affect cell-cycle kinetics	(a_G_,a_S_,a_M_,d_G_,d_S_,d_M_) = (a_G'_,a_S'_,a_M'_,d_G'_,d_S'_,d_M'_)
2	Labelling of S phase cells is instantaneous during the pulse phase	β = ∞
3	Labelled cells do not divide enough times during the experiment to shed label	α = 0
4	No dynamics perturbation under physiological conditions: steady-state cell number in each phase of cell cycle and in each cell population	(G_0_ = (G+G')_SS_,S_0_ = (S+S')_SS_,M_0_ = (M+M')_SS_) a_M_M+a_M'_M' = d_G_G+ d_S_S+ d_M_M+ d_G'_G'+ d_S'_S'+ d_M'_M'
5	No cell death or differentiation during S phase and G2/M phase	d_S_ = d_M_ = d_S'_ = d_M'_ = 0
6	Bio-disponibility of EdU is one hour after an injection	None
7	S phase lasts 6.5 hours	a_S_ = 1/6.5

Under our experimental conditions, the EdU labelling protocol is modelled by a first pulse phase (between t = 0 and t = 2 hours), a chase phase (between t = 2 and t = 16 hours) and a second pulse phase (between t = 16 and t = 16.5 hours). We defined an experimental result as a two-dimensional vector, representing the three percentages of G0/G1 unlabelled, G2/M unlabelled, and labelled cells which are equivalent to the G, M, and G’+S’+M’ in the model. Fitting to experimental data was done by simulating the progression of cells through the cell cycle and labelling over a bi-dimensional range of parameters. To constrain parameters, we used the biological hypotheses detailed in Materials and Methods (Table 3).

### Average proliferation rates and cell cycle durations in thymus and spleen from B6 and FVB mice

**Fig 4:**
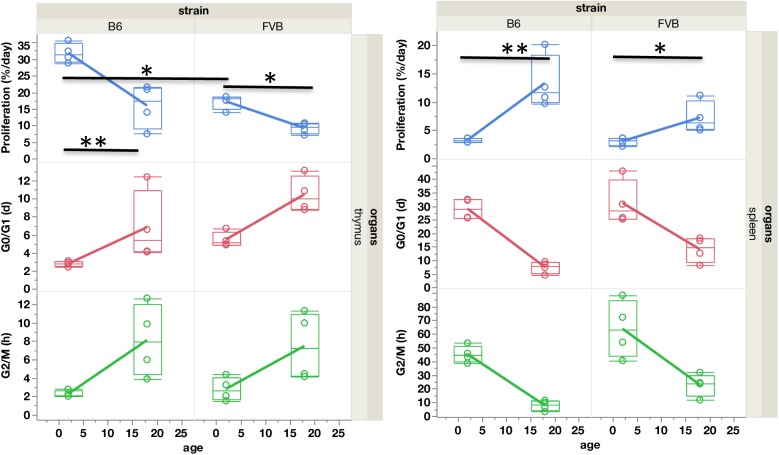
Proliferation rates and cell cycle phase durations in thymus and spleen. Proliferation rates (%/day), G0/G1 and G2/M phase durations (in days) in B6 and FVB mice (n = 4 per group) according to age (2 and 18 months) in whole thymus (left) and whole spleen (right). Values are indicated in the box plot, the median is the 50th percentile, the lower and upper limits of the box are the 25th and 75th percentiles and the whiskers indicate 1.5x(interquartile range). *: p<0.05. **: p<0.01. (See Materials and Methods section for statistical tests used). Statistics are for proliferation rates only. Mean values and SD are given in S1 Table.

We first compared proliferation rates for whole thymus and whole spleen, abstracting the phenotypic heterogeneity of T cells, in young and old B6 and FVB mice (Fig 4, S1 Table). In the thymus, proliferation rates can vary up to three-fold, from 32% per day in young B6 mice to 9% per day in old FVB mice. Proliferation rates of thymocytes in young B6 mice are in general twice the value of those in young FVB mice (p<0.05). The proliferation rates of thymocytes from old B6 mice and young FVB mice are similar (around 17%/day). In both strains, there is a two-fold drop of proliferation rates in thymus with aging (p<0.05 in spleen and p<0.01 in thymus). In the spleen, in contrast to the thymus, the proliferation rates increase with age. From about 3% per day in both young strains, it doubles in old FVB (p<0.05) and quadruples in old B6 mice (p<0.01).

The duration of G0/G1 phase, S, and G2/M gives an indication of the mean time between two cell cycles (inter-mitotic time), which can be calculated as 1/proliferation rate. Again, these values are heterogeneous; the duration of G0/G1 phase increases with aging, G2/M duration varies from 2 hours in young B6 thymocytes to up to 63 hours in young FVB splenocytes.

### Model validation: Simulation of another pulse-chase experiment from the literature

**Fig 5:**
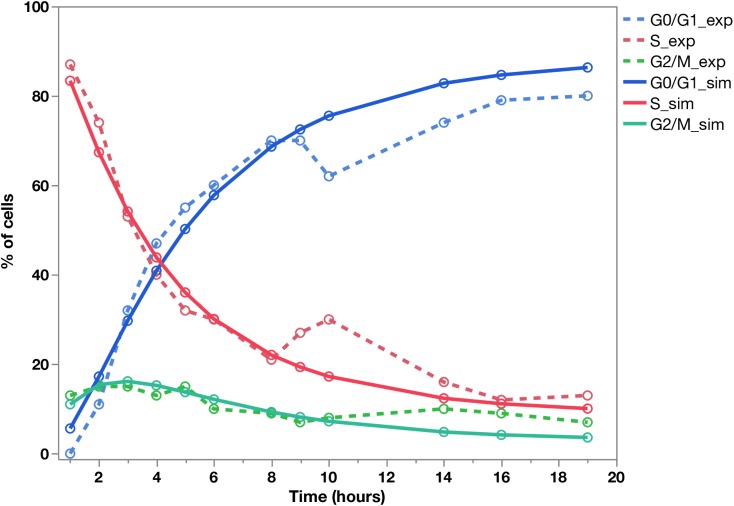
Validation of the mathematical model and parameter values with pulse/chase experimental data from [15]. Young B6 mice received a single BrdU pulse, and within the BrdU-labelled cells the percentages of cells in GO/G1, S or G2/M is recorded over a nineteen hour chase period. The points and dashed lines represent Baron experimental data. Simulation of the same pulse chase conditions using our mathematical model, with the mean parameter values (S1 Table) obtained from our 2-month-old B6 mice (n = 4). The points and continuous lines represent the results of our simulation.

In order to consolidate our results and validate our mathematical model, we simulated a different pulse/chase experiment, as performed in 1990 by Baron and Penit. This *in vivo* experiment also assesses whole thymocyte kinetics from six- to eight-week-old B6 mice, using a thymidine analogue Bromo-deoxyuridine (BrdU) uptake, versus DNA content measurement [15]. To simulate this experiment with our model, we set up a single injection of BrdU at the beginning of the experiment (1-hour pulse), followed by a nineteen-hour chase phase, during which within the BrdU-labelled cells the percentages of cells in G0/G1, S, and G2/M cells were quantified according to DNA content. We used cell cycle parameters retrieved from our own fitting procedure on the whole thymus of our 2-month old B6 mice (underlined in red in S1 Table), and we simulated the experiment by setting the initial state to be 100% of cells in S phase. We also set α = 0 and d_G_ = a_G_. The comparison of the experimental results provided by Baron and Penit [15] and the simulation of their pulse/chase protocol with our parameters obtained from B6 data is shown in Fig 5. The ability of our simulation to visibly approach Baron and Penit's experimental results confirms the plausibility of the biological parameters obtained in our young B6 mice and points to the fact that our model can be run with other experimental conditions of pulse/chase.

### Signature of proliferation according to T cell differentiation stage, age, and genetics

**Fig 6:**
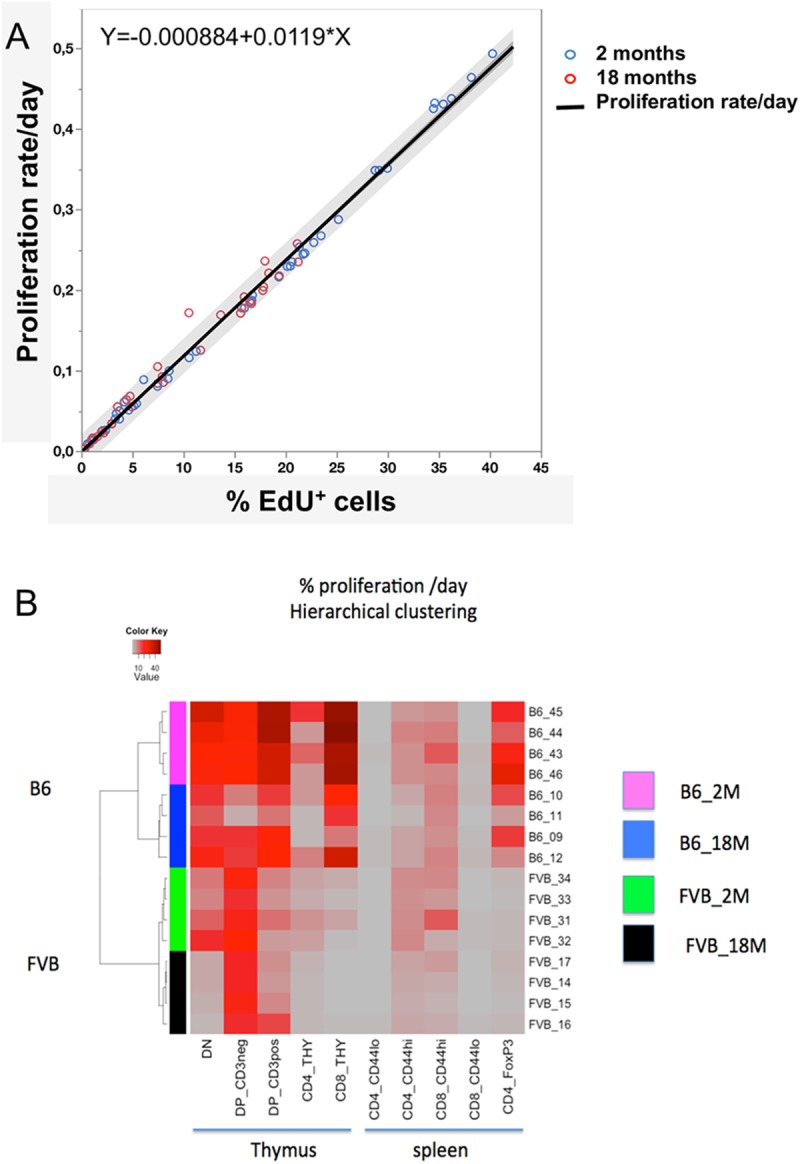
Signatures of lymphocyte proliferation according to strains and ages. The upper panel shows the correlation between the percentages of EdU^+^ cells observed 16 h after the pulse/chase/pulse experiment in thymus of B6 and FVB mice (80 values) and according to the age of mice (2 months: blue circle, 18 months: red circles). Hierarchical unsupervised clustering showing the clusters of mice according to the rates of proliferation. Values and statistics are given in S1, S2, S3 and S4 Tables. Proliferation rates/day are significantly different (p<0.05) comparing: **In thymus**: B6 2M vs B6 18M: 2M>18M in DP, CD8; FVB 2M vs FVB 18M: 2M>18M in DN and CD4; B6 2M vs FVB 2M:B6>FVB in DP CD3^+^, CD8; B6 18M vs FVB 18M:B6>FVB in CD8. **In spleen**: B6 2M vs B6 18M: 2M>18M in CD4 CD44^hi^; FVB 2M vs FVB 18M: 2M>18M in CD4 CD44^hi^;B6 2M vs FVB 2M:B6>FVB in CD4, CD4 Foxp3^+^; B6 18M vs FVB 18M:B6>FVB in CD4 Foxp3^+^, CD8 CD44^hi^

The complexity and heterogeneity of cell populations that compose the thymus led us to analyse, more deeply, the proliferation rates of cells according to their differentiation stages, thus across time. Thymocytes were further decomposed in the earliest immature triple negative thymocyte CD4^-^CD8^-^CD3^-^(TN), in DP (double positive thymocyte CD4+CD8+, sub-divided into DP CD3^lo^ and DP CD3^hi^ according to low or high expression of CD3), CD4, and CD8 mature CD3^+^ populations (S1 Fig upper panel). In the spleen, CD4 and CD8 T cells were separated as CD44^lo^ (naïve) and CD44^hi^ (antigen-experienced). CD4^+^Foxp3^+^ (regulatory T cells, Treg) cells were also identified as a sub-population of CD4 T cells (S1 Fig lower panel). From all data collected in the thymus, we were able to establish a linear correlation between the observed percentages of EdU^+^ cells and the fitted proliferation rates that is similar in the two strains of mice and is independent of the age (Fig 6). The equation y = -0.000884+0.0119x allows estimation of the proliferation rates, *y*, for further experiments using the same pulse/chase protocol duration, from *x* = %EdU^+^ cells (r^2^ = 0.99, p<0.01). Estimated parameter values are given in S2 Table.

Because the intragroup variability of proliferation rates is low while inter-group heterogeneity is high (S1 Fig), unsupervised hierarchical clustering allows identification of clusters of mice according to age, strain, and differentiation stage giving particular signatures (Fig 6). The heterogeneity of cell dynamics is revealed at the various steps of differentiation. If decomposition of the population is performed so as to identify rare populations as DN1 to DN4, then to DP that increase CD3 expression through maturation while modulating CD4 and CD8 co-expression, then one can observe oscillations in proliferation (S4 Table and S1 Fig median panel), as previously observed in young B6 mice using other protocols [36]. Hence in the thymus, while DN1 cells (the early thymic precursors) display minimal proliferation, in particular in old FVB mice (1.2%/day), CD8 mature thymocytes are the cells that display the highest proliferation rate (45%/day in young and a 2-fold decrease to 21%/day in old B6 mice) (S4 Table). Thus, the inter-mitotic time for CD8 CD3^+^ mature thymocytes in young B6 are of about 2 days (S2 Table), suggesting expansion of mature thymocytes before thymic export, while such proliferation is limited in FVB. The relative durations of G0/G1 and G2/M phase and thus inter-mitotic time increase with aging in most thymic populations (S2 Fig). In the spleen aging perturbations are more irregular. Altogether these durations are again heterogeneous according to the stage of differentiation and T cell lineage.

**Fig 7:**
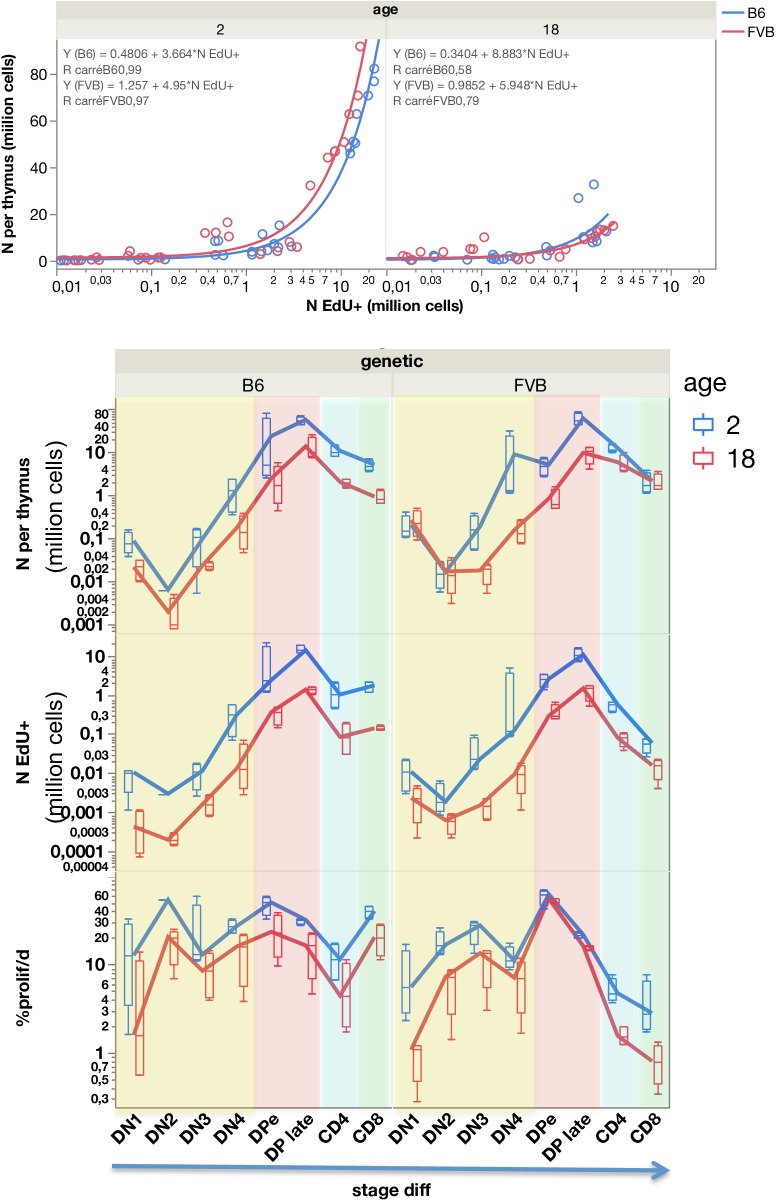
Quantification of dividing cells during thymocyte differentiation. For each stage of thymocyte differentiation, the number of cells and the number of EdU+ cells (in millions) is quantified by FACS after pulse/chase. The correlation between these numbers is shown according to strains and ages of mice (upper panel). Box plots represent the quartiles and the lines represent the evolution of the median values through stage of differentiation thus across time, in four mice per group (lower panel). DN CD3^-^ cells are in yellow, DP cells are in red and decomposed into DPearly (CD4^hi^CD8^hi^) and DP late (CD4^med^CD8^med^). Then DP cells differentiate either into CD4^+^CD3^+^ (blue) or into CD8^+^CD3^+^(green) mature T cells.

**Fig 8:**
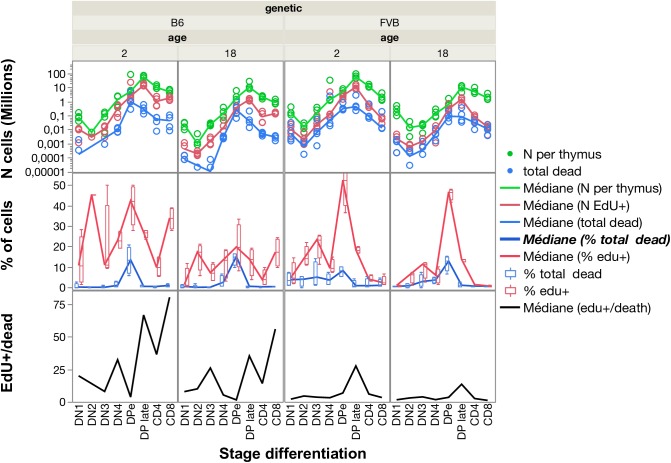
Quantification of cell proliferation and death in thymus. For each stage of thymocyte differentiation, the total number of live cells (green), of accumulated EdU^+^ (red) cells, and of dead cells (blue) after the pulse/chase/pulse are shown (upper panel). The percentage of EdU^+^ cells (red) and dead cells (blue) among each differentiation stage identified is given (lower panel). Quantification is done by FACS and given as the median (line) of 4 mice per group (points). In DN2 and DN3 cells in some B6 mice death is so rare that quantification is below 10 cells/thymus and sometimes up to 0 and cannot be represented on the log scale. The ratio of EdU^+^/dead cells gives a performance of cell expansion.

Numbers of cells per differentiation stage in the thymus are in accordance with the progression of thymocytes within 4 weeks as previously modelled in young FVB mice [1]. These numbers are correlated to the numbers of EdU^+^ cells (Fig 7 upper panel). Thus, an exponential growth of cells occurs during their residence in the thymus from DN2 where they actively divide up to late DP cells (Fig 7 lower panel). The minimal estimate of exponential cell divisions in B6 mice is 13 divisions in about 22 days, higher than the previous 9 divisions estimate [37]. This estimation does not take into account the cell death that occurs essentially at DN4 (1–3% of cells are dying/day) and at early DP stage for cells co-expressing the highest levels of CD4 and CD8 (death slightly increases from 13 to 15%/day in B6 mice and from 8 to 12%/day in FVB mice with ageing) during the thymic selection processes (Fig 7, S5 Table). We calculated the ratio of cell number EdU^+^/death as a performance index of T cell expansion. In FVB, the EdU^+^/death ratio is lower than in B6 at all stages of differentiation (S5 Table, Fig 8). Thus, although the total number of cells reaches similar expansion, the balance between division and death appears quite different in both strains.

When cells migrate from the thymus to the spleen they reduce their proliferation. Naïve CD44^lo^ T cells reduce their proliferation rate to <1%/day in all groups (S1 Fig, S3 Table, S4 Table). Thus a minimal inter-mitotic time of 226 days (a value similar to 218 days found in young B6 mice [27]), can be estimated for the more active CD8 naïve T cells in 2-month-old mice. In contrast, effector/memory T cells expressing CD44^hi^ after antigenic stimulation [38], are actively labelled, suggesting inter-mitotic times of 12 to 16 days in CD4 and CD8 from 2-month-old B6 while it increases about two-fold (32 and 37 days) in FVB old mice. Regulatory T cells are the T cell population with maximal proliferation rates in young B6 (21%/d), dividing every 5 to 12 days, respectively, in 2- and 18-month-old mice. In contrast, proliferation rates are as low as 1.4%/day suggesting that Treg cells divide only every 83 days in FVB mice, independently of the age.

## Discussion

Investigation of cell proliferation and turnover in space and time currently remains a challenge in immunology, in order to quantify the parameters of cell population dynamics *in vivo* according to tissue/organ and cell population heterogeneity, as well as to identify peculiarities related to the state of individuals, such as age and genetic origin [2, 3, 39–41].

### Experimental approach and mathematical modelling

The model developed here allows estimation of proliferation rates and inter-mitotic time (with estimated parameters and confidence intervals for G0/G1 and G2/M cell cycle phase durations as described in S1 Protocol and S6 Table) and allows quantitative comparison of the effect of age and genetics on lymphocyte dynamics from their early differentiation in thymus to their final maturation in spleen.

#### Heterogeneity of experimental approaches can be solved by modelling

Various experimental approaches, such as active cell labelling during cell cycle with nucleotide analogues, deuterated water or glucose, passive DNA content measurement, depletion of dividing cells, assessment of cyclin activity, and measurement at the level of populations or single cells, were recently reviewed [2]. Mathematical or computer modelling is necessary to interpret complex data and obtain standardized values for parameters such as proliferation rates and cell cycle phase durations. This is the only way to compare results issuing from different experimental approaches [2] (P. Loap et al. in preparation). BrdU, or more recently EdU labelling pulse-chase experiments, with various durations and routes of administration were used to investigate lymphocyte turnover and life-span in mice. Only a few reports concerned bi-dimensional analysis of BrdU/DNA content [15, 42], and these investigations were limited to whole organ cell populations. Indeed, sensitivity of fluorochromes to reveal BrdU labelling, and the small number of parameter investigations limited studies using old cytometers. In the last few years, the introduction of Clickit EdU technology and multi-colour flow cytometry has allowed investigation of cell phenotype together with EdU and DNA content labelling, as set up in this paper. Since complex experimental pulse chase periods change from one study to another, as in Baron's paper [15], the only way to interpret the results and to infer quantitative parameter values of proliferation per day is to develop a mathematical model.

Our mathematical model, designed with visual language accessible to biologists, is easy to manipulate to quantify lymphocyte dynamics parameters and can be used to simulate all kinds of pulse/chase experimental designs for further investigations. Moreover, it is flexible and can be easily modified to accommodate other phases of the cell cycle or different kinetics of label incorporation. This model was successfully applied to previous results from Penit group obtained with a different experimental pulse/chase protocol (cf Fig 5).

#### Cell heterogeneity and granularity of modelling

In the mathematical model and the process of fitting, it is assumed that the studied population has uniform cycling parameters. We make abstraction of certain important transition factors, such as differentiation, death, and migration between different compartments, as if the population was a closed system.

The estimated parameter values must be considered as averages over the whole population studied. Values for the total thymus are in fact an average over all sub-populations in the thymus, weighted by their relative importance in numbers of cells (DP cells are the most numerous intra-thymic population, and therefore results for the total thymus are similar to those for DP cells). A correct understanding of kinetic heterogeneity within the whole population therefore requires further identification into sub-populations so as to increase the resolution. However, precise rates of proliferation are difficult to calculate for heterogeneous populations during the differentiation process without integrating transition to cell death and transition between cell stages into a mathematic model. This is a general problem in the literature since to our knowledge there is currently no model that integrates such complex dynamical transitions. Indeed there is cell transition within populations (from rare DN, to DP to SP in thymus and from naïve to effector/memory in spleen), transition to cell death, and transition to another organ (from thymus to spleen) or recirculation. Here we can only quantify the numbers of dead cells at instant time (and not estimate rates/day) and death is not integrated in the current mathematical model. Inter-mitotic time and cell phase duration are only indicative but allow to compare the behaviour of various cell populations and conditions. While CD8 CD3^+^ mature thymocytes from young B6 mice have high proliferation rates (allowing for an inter-mitotic time of about 2.2 days) and possible expansion before thymic output, the CD8 CD3^+^ mature thymocytes from old FVB mice display a 100-fold lower proliferation rate and thus would be exported before having time to divide.

One should consider that between two pulses or within long pulse periods, cells may transit from differentiation stage or migrate to other tissues. However with a sixteen-hour long pulse/chase/pulse experiment these transitions are rather limited. The observation of a linear relationship between the number of cells labelled by EdU and the proliferation rate (Fig 6) is probably explained by the fact that as the duration of the experiment is smaller on average than the duration of one full cell cycle, labelled cells have not had the time to transit to another compartment, so that the number of labelled cells is globally proportional to the rate of entry into S phase and the proliferation rate, as mice are in steady-state. Nonetheless if we were to model a longer experimental protocol, it would undoubtedly be necessary to take into account these processes. Moreover, the parameter values observed with our sixteen-hour pulse/chase/pulse are confirmed by simulating the context of a single pulse followed by a nineteen-hour chase, as in the Baron paper that assessed whole thymocyte proliferation.

Considering these limitations, we believe that our experimental design and modelling is able to give, if not exact absolute values, at least a correct understanding of the relative differences between the heterogeneous populations studied.

#### Overlap of phases with DNA content analysis

Since DNA content only allows one to distinguish three phases in the cell cycle, it is not possible to give separate estimates of durations for G2 and M phases or, more problematically, for G0 and G1 phases. Thus G0/G1 phase duration is an average between G0 and G1 durations, weighted by their relative importance in numbers of cells. New protocols and modelling introducing Ki-67 labelling to separate G0 from G1 cells are under investigation (P. Loap et al. in preparation).

#### Parameter constraint and fit

The relatively low information content of the experimental dot plots (three proportions, i.e., a two-dimensional vector) constrains our search for parameters. We notably restrain ourselves to the steady-state case (Hyp.4) since we believe that the in vivo conditions of the experiment in normal mice justify this hypothesis. We also fix loss of cells in S and G2/M to be null (Hyp.5), as confirmed by observations [15]. Finally, we fix S phase duration to 6.5 hours (Hyp.7); this is a value that has been measured in the same type of cells, as simulated here [15]. A stretched cell cycle duration model [43] was recently proposed for mature T and B cells [44], but there is no indication this model could also occur in immature cells.

### Heterogeneity of lymphocyte dynamics according to cell population, age and genetic origin

The seminal work of Penit has addressed the quantification of proliferation in the thymus of young B6 mice [36]. However, to our knowledge we are the first to give proliferation rate/day at various granularities (from whole organ to rare populations) according to the time evolution of lymphocyte differentiation, from thymus to spleen, and in relation to the age and genetic origin of mice. FVB and B6 mice were chosen, because these strains belong to different phylogenetic clusters [45, 46] and display dissimilar T-Cell Receptor (TCR) repertoires as explained by chromosomal deletion of six Beta chain Variable region (BV) TCR families in FVB [47]. B6 and FVB mice differ in their lymphocyte and T cell compositions [48] and numbers in the various populations at steady-state [30]. Moreover, FVB mice display accelerated thymic involution and aging of VB repertoire as compared to B6 mice [30]. Thus, this suggests that variation in proliferation rates or cell death might explain these differences.

We have previously modelled thymocyte dynamics in young FVB mice and shown that 83% of the produced thymocytes die, with 35% of DP dying per day [1]. Here, we quantify thymocyte cell death as apoptotic cells in sub G0/G1 and as cells with a DNA content >4, corresponding to macrophages engulfing dying cells. While in total thymus about 2–3% of cells are in death at an instant time, major cell death occurs between DN3 and DN4 stages if VDJ recombination fails (3–5% of instant death) and at the DP stage by negligence and negative selection processes, with up to 15% instant cell death at early DP stages before CD4 and CD8 modulation. During death by neglect [49] and negative selection macrophages remove dying auto-reactive thymocytes, before cell membrane permeabilisation and chromatin condensation [50]. While the thymus of FVB young mice contains a higher number of cells than B6 mice (90 vs 81 millions of cells), we observe global defective proliferation rates as compared to B6 mice, in particular at early DN1 and DP stages but also for the proliferation of CD8 mature thymocytes (3%/day in FVB vs 40%/day in B6), and accumulation of cells at late DP stage. Conversely, in FVB mice, thymocyte death is generally higher. Thus, the death/EdU^+^ ratio is higher at all stages except in DP. [51]. Our results show that genetics and age influence T cell dynamics, in particular increase in the time spent in G0/G1 in thymus with aging, related to the G0 elongation theory of aging. Aging thus increases the inter-mitotic time, and contributes to the decrease of the total number of thymocytes as the individual ages. Impaired thymocyte proliferation and higher cell death in FVB mice could be related to the immaturity of their thymic epithelial cells [52] that normally interact with thymocyte during differentiation, selection, and migration, as recently modelled in B6 mice [35]. These defects in FVB mice may participate in the accelerated thymic involution and aging and the high CD4/CD8 ratio [30], also observed here (CD4/CD8 = 7 for FVB and 2 for B6). As modelled by Cohen’s group in a reactive animation [53], the cell competition for epithelial cells and decreased ratio of the dissociation rates of CD8^+^ and CD4^+^ thymocytes to 0.3, would induce an increase of the CD4/CD8 ratio to 7, thus possibly limiting the emergence of CD8 and their proliferation in FVB mice. As a consequence it could explain that while the recovery to steady state values following transient depletion of dividing cells is effective in young FVB mice [1], this process is defective in old FVB mice, leading to CD8 clonal expansions in periphery in spleen [30]. The defective Treg proliferation observed here in FVB mice with increased time passed in G0/G1 and G2/M even in young mice, may explain these TCR repertoire alterations in old mice. Indeed, in FVB mice, the transfer of Treg from young mice or the stimulation of their proliferation by low doses IL-2 treatment, initiated at 15 months of age, prevents repertoire alterations and CD8 clonal expansion at 2 years [30].

The global proliferation rate of splenocytes increases with aging from 3 to 13% per day in B6 and from 3 to 7% per day in FVB. This 2- to 4-fold increase in proliferation with aging reflects, however, considerable heterogeneity according to naïve or effector/memory T cells. Our previous modelling using transient depletion of dividing cells [1] led to the conclusion that “recent thymic emigrants” in the spleen, which represent around 50% of all naïve T cells in young mice, make 1 to 2 cell divisions during the 9 days after thymic output, allowing to complete the thymic clonal expansion (of positively selected T cells) up to obtain at least 2^4^ to 2^5^ cells per naïve cell clone. The mean proliferation rate of effector/memory T cells is 14 times that of naïve T cells. This high level of proliferation in effector memory antigen-experimented T cells (inter-mitotic time varies from 12 to 19 days in both strains) is in accordance with our previous results showing that immunological memory maintenance is dependent on active T cell division and that specific depletion of dividing T cells induces immunological amnesia while primary responses were intact [54]. However, with aging, the inter-mitotic time of CD44^hi^ cells increases. This suggests that the accumulation of CD44^hi^ cell number observed with aging is related to increased cell longevity rather than to active proliferation. This decrease in proliferation of effector memory T cells with aging could be related to hyper-glycosylation of T cell surface macromolecules, leading to altered T cell signalling [55]. Altered signalling pathways occur in T cells with aging, in particular as alteration of lipid raft polarisation, altering early steps of T cell activation, with increased activity of SHP-1 acting as a negative feedback on lymphocyte proliferation [56, 57]. This could explain the altered vaccine response in old individuals, and should be considered in systems biology approaches for vaccine design [58].

The CD4^+^Foxp3^+^ regulatory T cells, which represent less than 10% of CD4 splenic T cells, display the highest proliferation rate within splenic T cells. This is in accordance with their peculiar selection by medullary thymic epithelial cells [59] presenting promiscuous antigens because of Auto-Immune REgulator (AIRE) expression [60] of autoreactive Treg, then triggered in peripheral tissues to division by self-antigens cognition. We have previously shown that dominant tolerance to allogeneic grafts is related to antigen specific Treg cells selection in thymus [61]. In fact, a delicate balance between proliferation or suppressive function of Treg is of importance to insure the negative feedback loop, which controls the proliferation of other T cells. When Treg cells are under “suppressive mode”, they are anergic, cannot proliferate [62] and thus less sensitive to specific depletion of dividing T cells than other CD4 T cells. Thus, specifically targeting cells in proliferation can induce dominant tolerance [51].

Our general conclusion is that FVB mice have lower proliferation rates than B6 mice, and that with aging T lymphocytes have lower proliferation rates, although all mice are kept in the same environment. This certainly contributes to an accelerating aging. This reveals a specific “signature” of proliferation across differentiation stages in each of the two strains. This is in favour of the influence of genetic background and age on T cell dynamics and thus on the homeostatic equilibrium of the immune system. Therefore, the sensitivity of dividing cells—to depletion treatment by conditional immuno-pharmaco-genetics that also reveal cell dynamics [1, 30, 51, 54] or to gene therapy where retrovirus integration requires cell division [63]—could be very different with aging and genetic origin. Cell proliferation, age and genetics then influence the induction or control memory [54] and induction of tolerance [30, 51]. Thus, knowledge on lymphocyte dynamics and proliferation of sub-populations is of importance for clinical applications.

## Materials and methods

### Mice

C57BL/6/N (B6) and FVB/N (FVB) mice were obtained from Charles River Laboratories maintained in SPF conditions and used at 2 and 18 months of age. Mice were manipulated according to European council directive 86/609/EEC of 24 November 1986 and with the approval of an ethics committee.

### In vivo EdU treatment and multicolor flow cytometry analysis

The experimental protocol is summarized in Fig 1. Mice received three intra-peritoneal injections of 1 mg of EdU (5-ethynyl-2'-deoxyuridine, Life technologies) at 0, 1 and 16 hours and were killed half an hour after the last injection to remove thymus and spleen. Cell suspensions were obtained by mechanical disruption of organs in PBS + 3% newborn calf serum at 4°C, were then washed and submitted to CD16/CD32 blockage with 2.4G2 hybridoma cell supernatant for 10 minutes. Cell membrane labeling was then done for 20 minutes with antibodies coupled with fluorochromes Percp, APC-H7, and APC. After washing, the cells were fixed and permeabilized with the ClickIT fixative, and EdU was revealed by Click-IT containing Alexa488-azide. The last step consisted of staining with antibodies labeled with PE and PE-cy7 for 30 minutes and washing with Perm/Wash buffer (Becton-Dickinson). 1 μL FxCycle violet stain (Life Technologies) was added per tube, 30 minutes before acquisition. Cell parameters were acquired on LSR2 equipped with 405, 488, and 633 nm lasers (Becton-Dickinson) at a rate of 2000 cells/sec with DIVA software. Cells were analysed with Flowjo (www.flowjo.com) on the basis of structural cell markers FSC-A (Forward Scatter-Area), SSC-A (Side Scatter-Area), FSC-W (Forward Scatter-Width) to remove debris, apoptotic cells, and doublets. Multicolour expression of specific markers CD3, CD4, CD8, CD25, CD44 allowed identification of cell sub-population phenotype by hierarchical gating. Then, DNA content and EdU label enabled construction of bi-dimensional dot plots to determine cell kinetics in each population. Dead cells were quantified in subG0/G1 apoptotic cells and cell that display a DNA content >4N, corresponding to macrophages engulfing dying cells before apoptosis.

### Mathematical model

We designed an ODE model describing cell dynamics during an EdU labelling experiment. Cells cycle through G0/G1, S, G2/M and are either unlabelled or labelled by EdU, giving six populations of cells represented by the six following ODEs (system of Eq (1)):

$$\begin{array}{l} \frac{dG}{dt}=2a_{M}M+2\alpha a_{M\prime }M^{\prime }-(a_{G}+d_{G})G\\ \frac{dS}{dt}=a_{G}G-(a_{S}+d_{S})S-[0,\beta ]S\\ \frac{dM}{dt}=a_{S}S-(a_{M}+d_{M})M\\ \frac{dG\prime }{dt}=2(1-\alpha )a_{M^{\prime }}M\prime -(a_{G^{\prime }}+d_{G^{\prime }})G\prime \\ \frac{dS\prime }{dt}=a_{G\prime }G^{\prime }-\left(a_{S^{\prime }}+d_{S^{\prime }}\right)S^{\prime }+\left[0,\beta \right]S\\ \frac{dM\prime }{dt}=a_{S\prime }S\prime -\left(a_{M^{\prime }}+d_{M^{\prime }}\right)M\prime \end{array}$$ (1)

Populations are described in Table 1, parameters in Table 2, the equivalent state transition diagram is in Fig 2.

G, S, M are numbers of cells in G0/G1, S, G2/M phase of the cell cycle, respectively. G’, S’, M’ are the corresponding EdU labelled cell numbers. a_X_ terms correspond to rates of entry into the next phase of cells in X phase (X being either G, S, M or G’, S’, M’). The exit of cells, either due to death, differentiation, or migration is represented by d_X_ terms. EdU labelling intensity is modelled by two terms: β is the rate of labelling of S phase cells by EdU, while α is the rate of loss of labelling by labelled cells after division. To distinguish pulse and chase phases of the experiment, we have introduced the symbol [0,β], meaning that during the chase phase (no EdU) we use 0, while during the pulse phase we use β in the equation (equivalent to a Dirac function multiplied by β, with the Dirac function equal to 1 during pulse and 0 during chase). The proliferation rate is defined as p = a_M_M+a_M'_M', i.e., it is the number of cells in G2/M phase, unlabelled (M) and labelled (M'), which divide at rate a_M_ (unlabelled) or a_M'_ (labelled).

A simulation is run by assigning: the twelve parameters of the cell cycle (a_G_,a_S_,a_M_,d_G_,d_S_,d_M_) and (a_G'_,a_S'_,a_M'_,d_G'_,d_S'_,d_M'_); the two parameters corresponding to de-labelling and labelling, respectively (α,β); the initial state of the cell populations (G_0_,S_0_,M_0_) and (G'_0_,S'_0_,M'_0_); and the labelling protocol, i.e., the beginning and end of pulse and chase phases.

### Fit of simulations to experimental results

The results of simulations were fitted to experimental results represented by EdU/DNA flow cytometry dot plots. On the dot plot, three groups of cells can be individualized and their relative percentages determined according to EdU labelling and DNA content: G0/G1 EdU^-^, G2/M EdU^-^, and EdU^+^. We therefore defined an experimental result by three percentages: (G_exp_,M_exp_,(G'+S'+M')_exp_). To fit our model to experimental results and to restrict the search for parameters, we made hypotheses listed in Table 3:

- Hyp.1: Parameters during pulse and chase are equal, reducing to six the number of parameters of the cell cycle, i.e., (a_G_,a_S_,a_M_,d_G_,d_S_,d_M_) = (a_G'_,a_S'_,a_M'_,d_G'_,d_S'_,d_M'_).
- Hyp.2: EdU cell labelling is instantaneous, i.e., β = ∞. While mathematically not integrable as such, this is very simply implemented in the computer model by transferring at the start of the pulse phase all unlabelled S cells to the labelled S compartment, and by allowing the unmarked G1 cells to only go into the labelled S compartment during pulse phase.
- Hyp.3: There is no shedding of EdU label during the time of the experiment, i.e., α = 0.
- Hyp.4: Cells are in a steady-state, and so is the distribution of cells in each cycle phase ((G+G')_SS_,(S+S')_SS_,(M+M')_SS_) as described by system of Eq (2):

$$\left\{ \begin{array}{c} \frac{d\left(G+G^{\prime }\right)}{dt}=0\\ \frac{d\left(S+S^{\prime }\right)}{dt}=0\\ \frac{d\left(M+M^{\prime }\right)}{dt}=0 \end{array}\right.\Leftrightarrow \left\{ \begin{array}{c} {\left(G+G\prime \right)}_{ss}=\frac{200a_{M}\left(a_{S}+d_{S}\right)}{2a_{M}\left(a_{S}+d_{S}\right)+2a_{M}a_{G}+\left(a_{G}+d_{G}\right)\left(a_{S}+d_{S}\right)}\\ {\left(S+S\prime \right)}_{ss}=\frac{200a_{M}a_{G}}{2a_{M}\left(a_{S}+d_{S}\right)+2a_{M}a_{G}+\left(a_{G}+d_{G}\right)\left(a_{S}+d_{S}\right)}\\ {\left(M+M\prime \right)}_{ss}=\frac{100\left(a_{G}+d_{G}\right)\left(a_{S}+d_{S}\right)}{2a_{M}\left(a_{S}+d_{S}\right)+2a_{M}a_{G}+\left(a_{G}+d_{G}\right)\left(a_{S}+d_{S}\right)} \end{array}\right.$$ (2)

with (*G* + *G*′)_*SS*_ + (*S* + *S*′)_*SS*_ + (*M* + *M*′)_*SS*_ = 100(%).

As a consequence, the total number of cells during the experiment is constant and percentages can be used. This also gives a conservation equation as follows:

a_M_M+a_M'_M' = d_G_G+ d_S_S+ d_M_M+ d_G'_G'+ d_S'_S'+ d_M'_M'.

- Hyp.5: There is no exit during S or G2/M phase [15], i.e., d_S_ = d_M_ = d_S'_ = d_M'_ = 0.
- Hyp.6: One injection of EdU is modelled by a one-hour pulse [32].
- Hyp.7: S phase lasts 6.5 hours [15], i.e., a_S_ = 1/6.5.

With these hypotheses, ten out of the twelve parameters of the cell cycle are fixed, except for a_M_ and a_G_, since:

(a_G_,a_S_ = 1/6.5,a_M_,d_G_ = a_G_,d_S_ = 0,d_M_ = 0) = (a_G'_,a_S'_,a_M'_,d_G'_,d_S'_,d_M'_). The two parameters of EdU labelling intensity are: (α = 0,β = ∞). The initial state is:

(G_0_ = (G+G')_SS_,S_0_ = (S+S')_SS_,M_0_ = (M+M')_SS_,G'_0_ = 0,S'_0_ = 0,M'_0_ = 0).

To fit our model to experimental results, we run simulations over a two-dimensional range of parameters (a_G_,a_M_). The time step chosen with the integration tool was 0.01 hour (36 seconds). The best fit is the couple of parameters which minimizes the Euclidean distance between (G_exp_,M_exp_,(G'+S'+M')_exp_) and (G_sim_,M_sim_,(G'+S'+M')_sim_).

All results obtained have a Euclidean distance to experiment of less than 0.05. We deduce from the best fit the mean duration of G0/G1 phase 1/a_G_, the mean duration of G2/M phase 1/a_M_, and the proliferation rate p, which with our hypotheses is equal to 200a_M_a_G_/(2a_M_+13a_M_a_G_+2a_G_) (in percentage of cells dividing per hour).

The identifiability of parameters obtained during the fitting procedure and some illustrative results are given in S1 Protocol and S6 Table. We also give standard deviations for our estimates of proliferation rates in total thymus and spleen, calculated with the Hessian matrix (variance-covariance matrix method).

### Statistics

Statistics were performed using the R software (www.rproject.org). Statistical significance level was fixed at α = 0.05 (Type 1 error). Before applying comparison tests, all data were assessed for normality by Shapiro-Wilk’s test and for equality of variances by Levene’s test. These results led us to the following choice of statistical tests: (i) if the normality hypothesis did not hold, we used the non-parametric Mann-Whitney test (ii) otherwise, we used the Student's two-tailed unpaired T-test with a hypothesis of either equal variances or unequal variances, depending on the result of Levene’s test. We computed arithmetical means and standard deviations (variance square root) for each group (n = 4).

### Software

The mathematical model of cell proliferation and its fitting to experimental data were developed and simulated using Python (http://www.python.org). To allow high-throughput analyses, we developed a program in Python to implement the fitting procedure on tables of values extracted from the dot plot manual gates with FlowJo software or from published results recovered from 2-dimentional descriptions of cell kinetics. Graphs were produced with JMPpro11.

## Supporting information

S1 Fig Heterogeneity of proliferation according to strain, age, organ and T cell population. These values are only indicative, since transition of cells from one stage to another and transition to death are not modelled. Each line represents the values obtained for one mouse. The means of these values are given in S1 to S4 Tables.
(TIF)

S2 Fig Heterogeneity of duration of cycle phase and intermitotic time in thymic and splenic sub-populations, and variability with strains of mice and aging. The G0/G1 and G2/M estimated durations are in hours. The inter-mitotic time durations are given in days. The lines represent median values of 4 mice per group.
(TIFF)

S1 Table Parameter values obtained by fitting the mathematical model to whole spleen and whole thymus. Mean and standard deviation with n = 4 mice per group
(TIF)

S2 Table Thymocyte dynamics parameter values in B6 and FVB mice aged 2 and 18 months. For each differentiation stage, the mean number of cell/thymus (n = 4) and standard deviation is given. TN corresponds to immature triple negative cells CD4^-^CD8^-^ CD3^-^ cells. DP CD3^-^ and DP CD3^+^ stages are the decomposition of DP cells. The percentage of labelled cells/16h and the percentage of proliferation/day are correlated, as shown in Fig 7. Estimated duration of G0/G1 and G2/M are given in days. The duration of S phase is fixed to 6.5 hours. Total time indicates hypothetical inter-mitotic time (1/proliferation rate). These values are only indicative, since transition of cells from one stage to another and to death are not modelled. **Statistical analysis obtained by fitting the procedure for the proliferation rate (%/day) in thymus.** Statistics are given for the populations of total thymus, DN CD3^-^ (TN); DP CD3^lo^, DP CD3^hi^, CD4^+^CD3^+^ and CD8^+^CD3^+^ thymocytes. B6_2M: 2 month-old B6 mice, B6_18M: 18 month-old B6 mice, FVB_2M: 2 month-old FVB mice, FVB_18M: 18 month-old FVB mice. Level of significance of statistical tests: > (resp. <) indicates that in population p, group a (on the left) has a mean which is superior (resp. inferior) to group b (on the right) with a level of significance of p<0.05; >> (resp. <<) is for p<0.01; >>> (resp. <<<) is for p<0.001.
(TIF)

S3 Table Splenocyte dynamics parameter values in B6 and FVB mice aged 2 and 18 months. Estimated percentage of proliferation/day from the model, allowing estimation of duration of G0/G1, G2/M phase, and the potential inter-mitotic time for various cell populations. Whole CD4-CD3^hi^ and CD8-CD3^hi^ cells are decomposed into CD44^hi^ (effector/memory) and CD44^lo^ (naïve) cells showing the heterogeneity of dynamics according to the granularity of populations. CD4-Foxp3 are regulatory T cells Foxp3^hi^. These values are only indicative, since transition of cells from one stage to another and to death are not modelled. **Statistical analysis obtained by fitting the procedure for the proliferation rate (%/day), in spleen:** Statistics are given for the populations of total spleen; CD4, CD4 CD44^lo^, CD4 CD44^hi^, CD4 FoxP3+; CD8, CD8 CD44^lo^ and CD8 CD44^hi^ splenocytes. B6_2M: 2 month-old B6 mice, B6_18M: 18 month-old B6 mice, FVB_2M: 2 month-old FVB mice, FVB_18M: 18 month-old FVB mice. **Level of significance of statistical tests:** > (resp. <) indicates that in population p, group a (on the left) has a mean which is superior (resp. inferior) to group b (on the right) with a level of significance of p<0.05; >> (resp. <<) is for p<0.01; >>> (resp. <<<) is for p<0.001.
(TIF)

S4 Table Comparison of proliferation rates according to strain, age, organ, and T cell populations. Total represents the whole organ. CD4 and CD8 mature T cells are observed in the thymus and spleen. Mean proliferation rates per day are given during the transition of cells from DN1 to DP_CD3^+^ then CD4 or CD8 in thymus and from naïve (CD44^lo^) to effector/memory (CD44^hi^) differentiation in spleen; Foxp3 cells are a subpopulation of CD4 that are CD44^hi^. These values are only indicative, since the transition of cells from one stage to another and to death are not modelled.
(TIF)

S5 Table Evolution of thymocyte numbers according to strains, ages, and differentiation stages. Numbers are given as millions of cells in the thymus (see Figs 7 and 8). The ratio between EdU^+^ and dead cells gives a performance of cell expansion. DP^total^ represents the sum of the DPe (gated on CD4^hi^CD8^hi^) and DP^late^ (gated on CD4^med^CD8^med^).
(TIF)

S6 Table Proliferation rates with confidence intervals and standard deviations of single mice, for total thymus and total spleen. Proliferation rates (%/day) with confidence intervals (CI = IC_min—IC_max) and standard deviations (sd) calculated with the use of the Hessian matrix of all sixteen mice in whole thymus and whole spleen. Confidence intervals and standard deviations are calculated as described in the S1 Protocol.
(TIF)

S1 Protocol Identifiability of parameters and calculation of confidence intervals and standard deviations in individual mice allowing for mathematical proliferation rate and cell cycle phase duration estimation. (DOCX)
