# Supplementary figures and images for: Modelling T cell proliferation: Dynamics heterogeneity depending on cell differentiation, age, and genetic background
Source: PLoS Comput Biol. 2017 Mar 13;13(3):e1005417. doi: 10.1371/journal.pcbi.1005417 (PMC5367836; doi:10.1371/journal.pcbi.1005417)

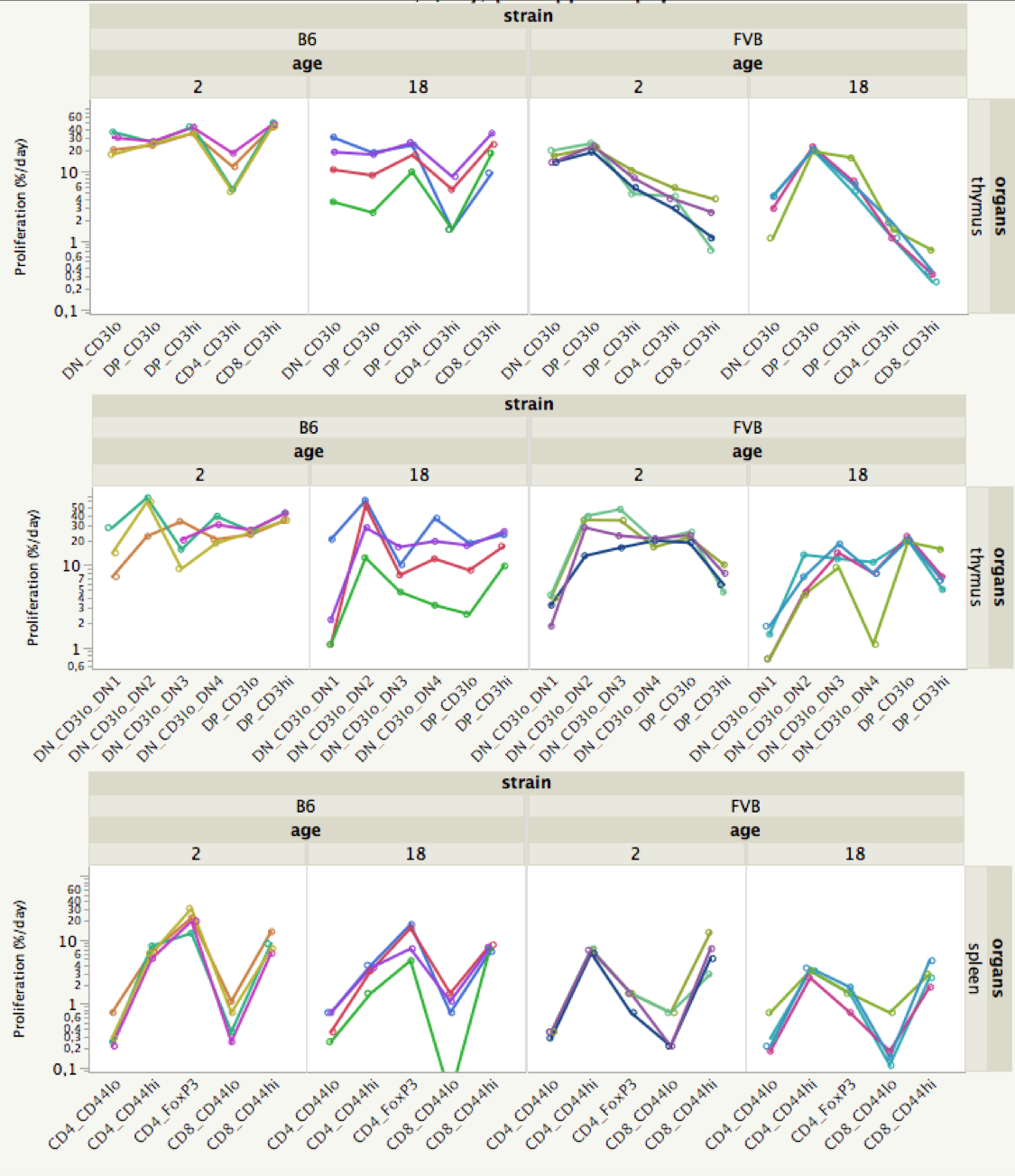

Supplement: S1 Fig — These values are only indicative, since transition of cells from one stage to another and transition to death are not modelled. Each line represents the values obtained for one mouse. The means of these values are given in S1 to S4 Tables. (TIF) [file pcbi.1005417.s001.tif]

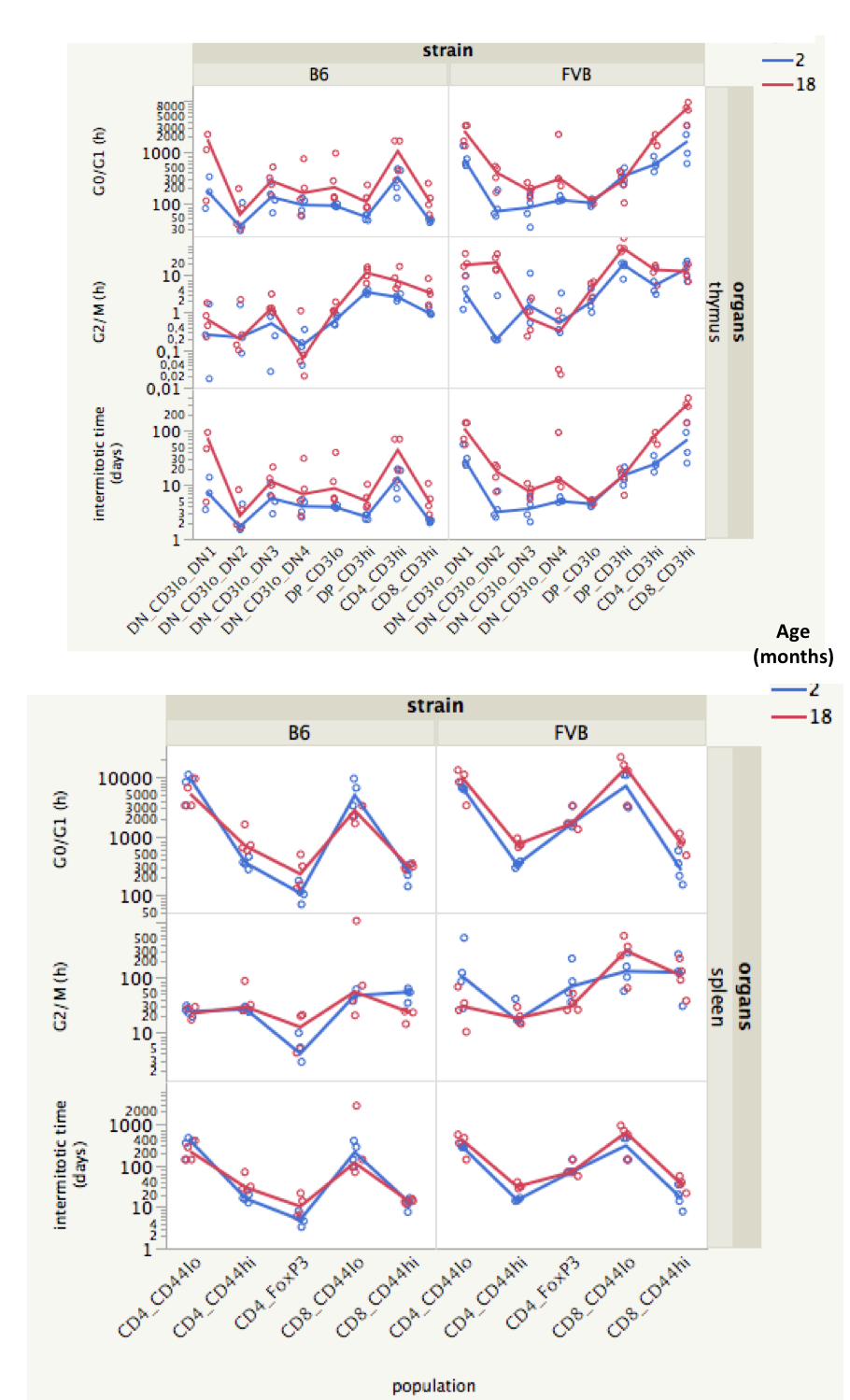

Supplement: S2 Fig — The G0/G1 and G2/M estimated durations are in hours. The inter-mitotic time durations are given in days. The lines represent median values of 4 mice per group. (TIFF) [file pcbi.1005417.s002.tiff]

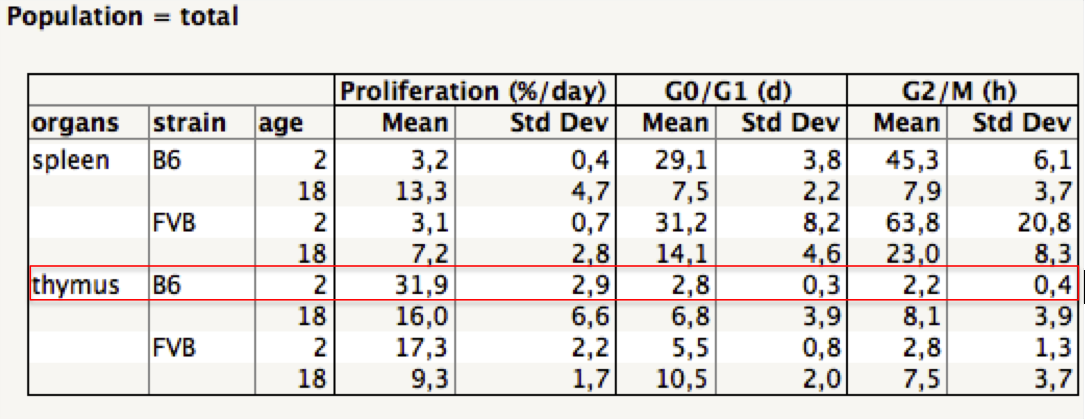

Supplement: S1 Table — Mean and standard deviation with n = 4 mice per group (TIF) [file pcbi.1005417.s003.tif]

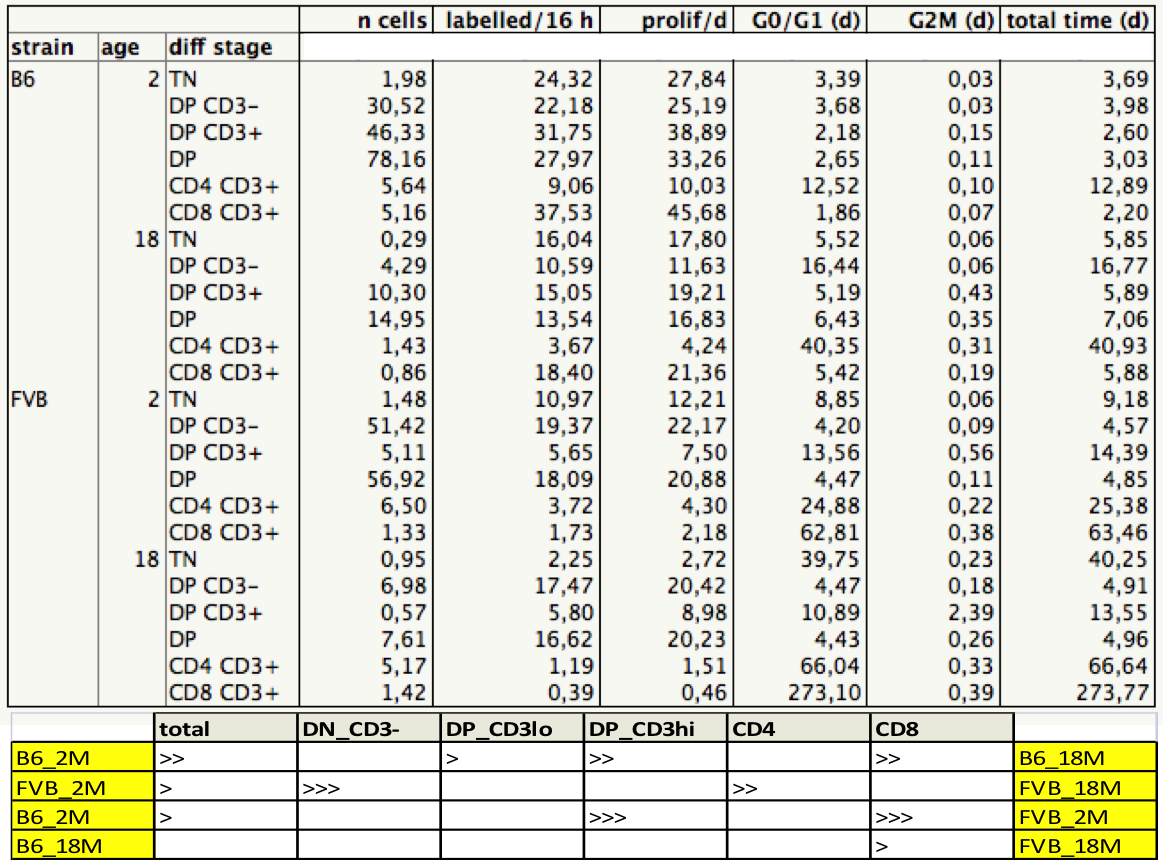

Supplement: S2 Table — For each differentiation stage, the mean number of cell/thymus (n = 4) and standard deviation is given. TN corresponds to immature triple negative cells CD4-CD8- CD3- cells. DP CD3- and DP CD3+ stages are the decomposition of DP cells. The percentage of labelled cells/16h and the percentage of proliferation/day are correlated, as shown in Fig 7. Estimated duration of G0/G1 and G2/M are given in days. The duration of S phase is fixed to 6.5 hours. Total time indicates hypothetical inter-mitotic time (1/proliferation rate). These values are only indicative, since transition of cells from one stage to another and to death are not modelled. Statistical analysis obtained by fitting the procedure for the proliferation rate (%/day) in thymus. Statistics are given for the populations of total thymus, DN CD3- (TN); DP CD3lo, DP CD3hi, CD4+CD3+ and CD8+CD3+ thymocytes. B6_2M: 2 month-old B6 mice, B6_18M: 18 month-old B6 mice, FVB_2M: 2 month-old FVB mice, FVB_18M: 18 month-old FVB mice. Level of significance of statistical tests: > (resp. <) indicates that in population p, group a (on the left) has a mean which is superior (resp. inferior) to group b (on the right) with a level of significance of p<0.05; >> (resp. <<) is for p<0.01; >>> (resp. <<<) is for p<0.001. (TIF) [file pcbi.1005417.s004.tif]

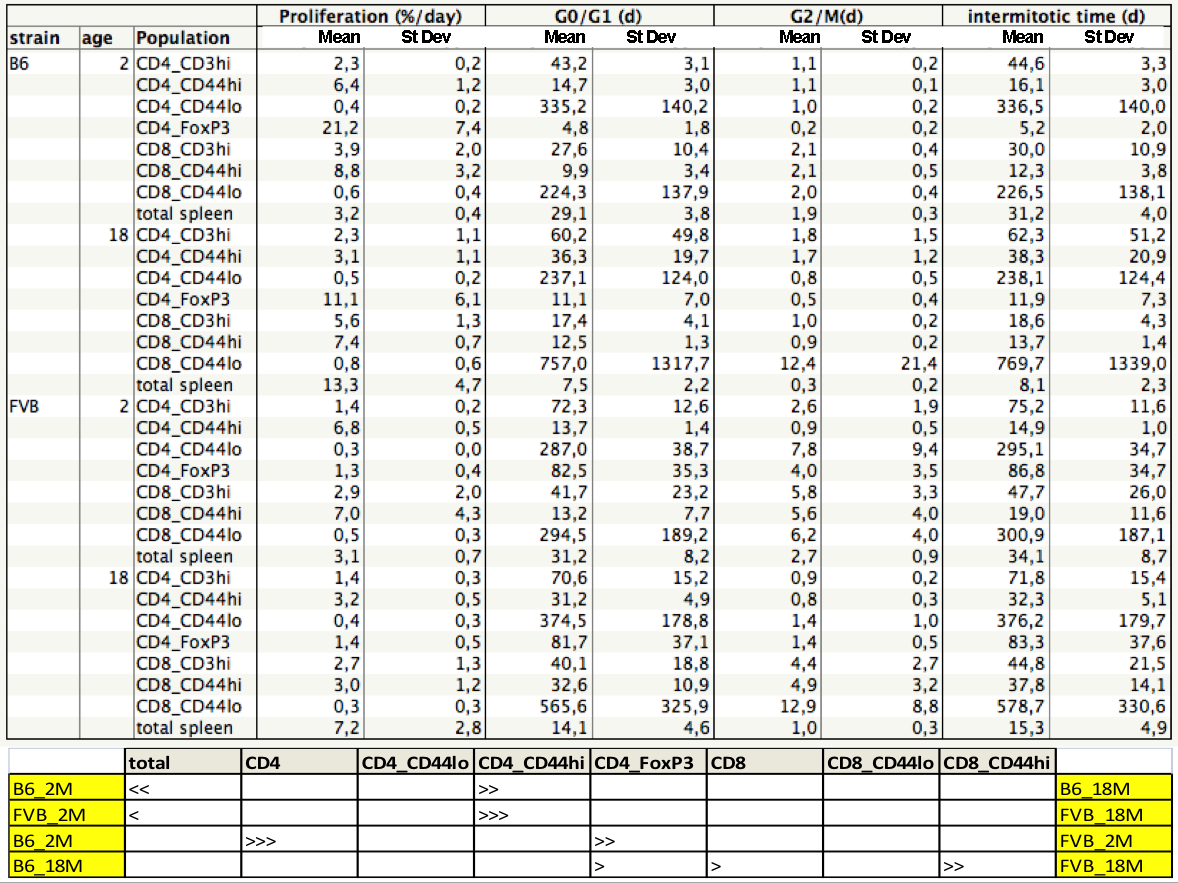

Supplement: S3 Table — Estimated percentage of proliferation/day from the model, allowing estimation of duration of G0/G1, G2/M phase, and the potential inter-mitotic time for various cell populations. Whole CD4-CD3hi and CD8-CD3hi cells are decomposed into CD44hi (effector/memory) and CD44lo (naïve) cells showing the heterogeneity of dynamics according to the granularity of populations. CD4-Foxp3 are regulatory T cells Foxp3hi. These values are only indicative, since transition of cells from one stage to another and to death are not modelled. Statistical analysis obtained by fitting the procedure for the proliferation rate (%/day), in spleen: Statistics are given for the populations of total spleen; CD4, CD4 CD44lo, CD4 CD44hi, CD4 FoxP3+; CD8, CD8 CD44lo and CD8 CD44hi splenocytes. B6_2M: 2 month-old B6 mice, B6_18M: 18 month-old B6 mice, FVB_2M: 2 month-old FVB mice, FVB_18M: 18 month-old FVB mice. Level of significance of statistical tests: > (resp. <) indicates that in population p, group a (on the left) has a mean which is superior (resp. inferior) to group b (on the right) with a level of significance of p<0.05; >> (resp. <<) is for p<0.01; >>> (resp. <<<) is for p<0.001. (TIF) [file pcbi.1005417.s005.tif]

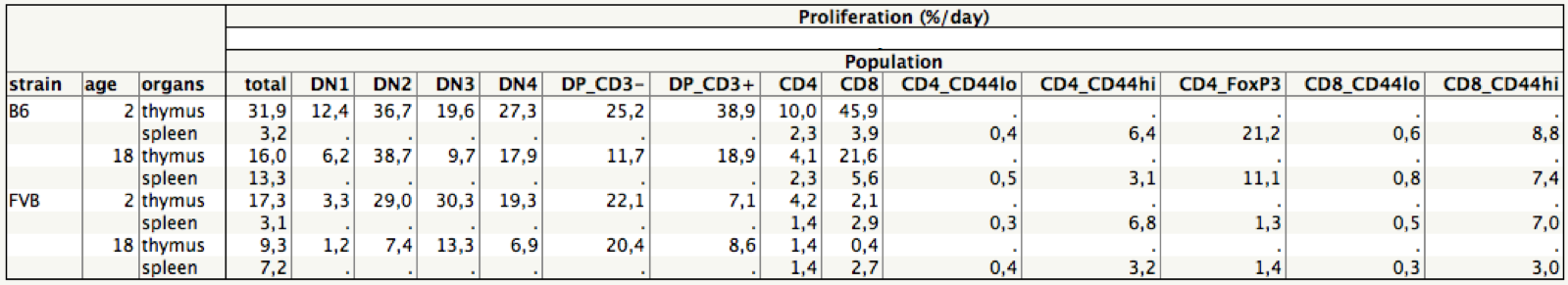

Supplement: S4 Table — Total represents the whole organ. CD4 and CD8 mature T cells are observed in the thymus and spleen. Mean proliferation rates per day are given during the transition of cells from DN1 to DP_CD3+ then CD4 or CD8 in thymus and from naïve (CD44lo) to effector/memory (CD44hi) differentiation in spleen; Foxp3 cells are a subpopulation of CD4 that are CD44hi. These values are only indicative, since the transition of cells from one stage to another and to death are not modelled. (TIF) [file pcbi.1005417.s006.tif]

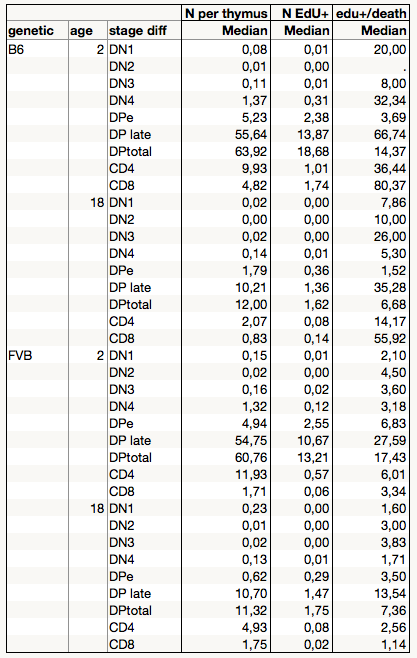

Supplement: S5 Table — Numbers are given as millions of cells in the thymus (see Figs 7 and 8). The ratio between EdU+ and dead cells gives a performance of cell expansion. DPtotal represents the sum of the DPe (gated on CD4hiCD8hi) and DPlate (gated on CD4medCD8med). (TIF) [file pcbi.1005417.s007.tif]

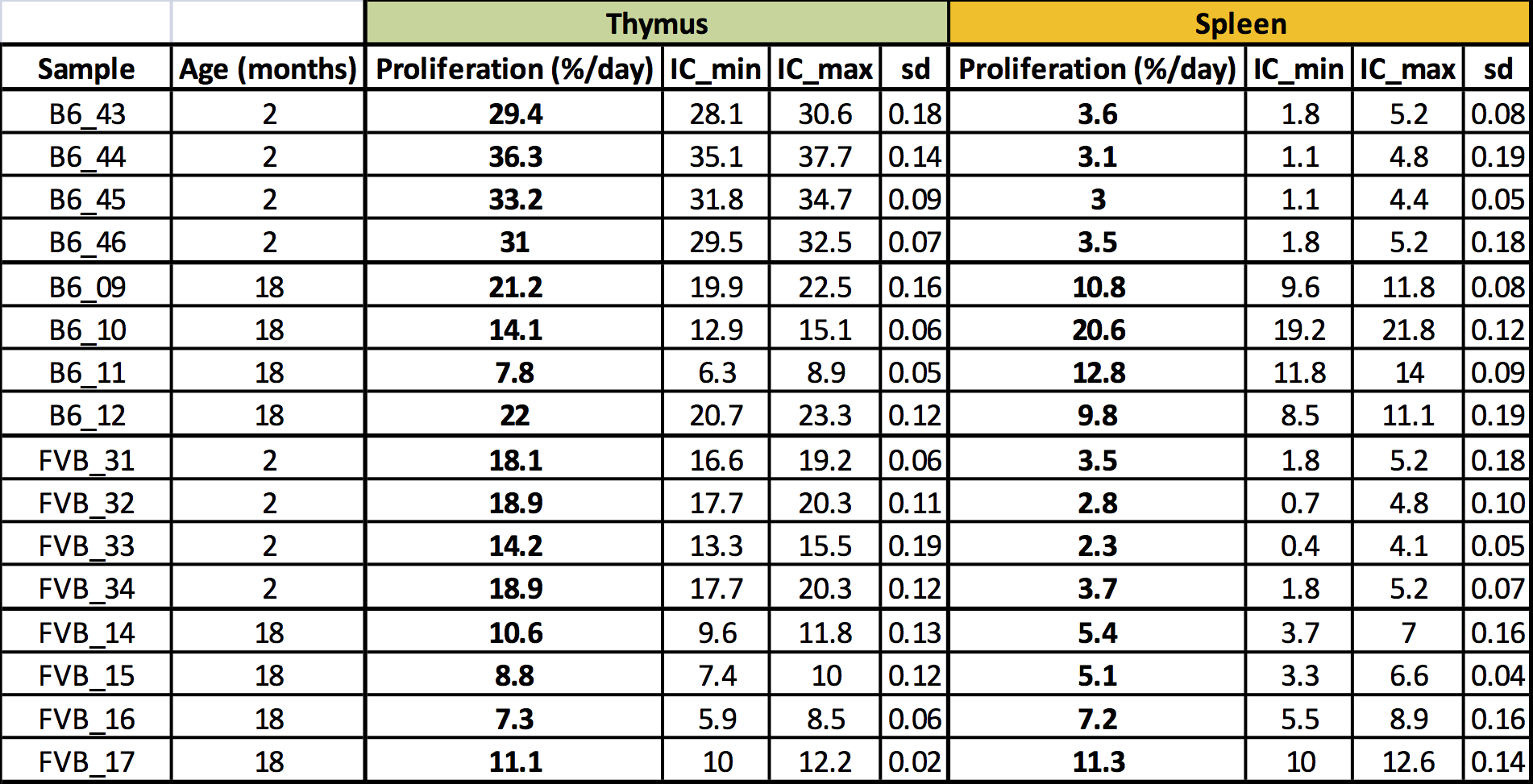

Supplement: S6 Table — Proliferation rates (%/day) with confidence intervals (CI = IC_min—IC_max) and standard deviations (sd) calculated with the use of the Hessian matrix of all sixteen mice in whole thymus and whole spleen. Confidence intervals and standard deviations are calculated as described in the S1 Protocol. (TIF) [file pcbi.1005417.s008.tif]
