# Supplementary material for: Modelling T cell proliferation: Dynamics heterogeneity depending on cell differentiation, age, and genetic background
Source: PLoS Comput Biol. 2017 Mar 13;13(3):e1005417. doi: 10.1371/journal.pcbi.1005417 (PMC5367836; doi:10.1371/journal.pcbi.1005417)
Supplement: S1 Protocol — (DOCX) [file pcbi.1005417.s009.docx]

**S1 Protocol: Identifiability of parameters and calculation of confidence intervals and standard deviations in individual mice allowing for mathematical proliferation rate and cell cycle phase duration estimation**

**
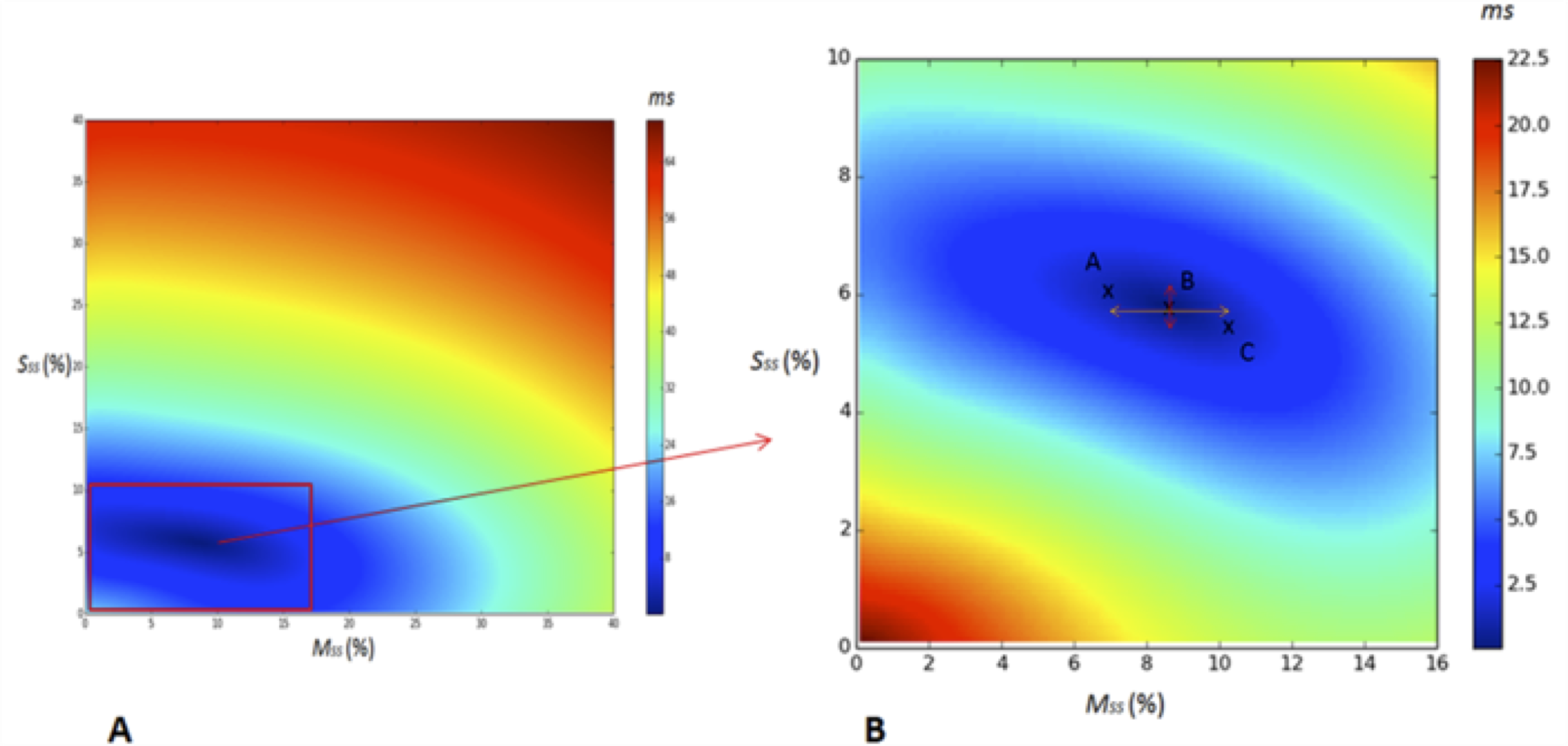
**

**Identifiability of parameters obtained during the fitting procedure, with a** **graphical representation of the fitting procedure.** The example is for total thymocytes of a B6 18-month old mouse. After having run 160,000 simulations (S_SS_ and M_SS_ each ranging from 0.1% to 40% every 0.1%), the best fit is the pair of parameters which minimizes the Euclidean distance between experiment and simulation. Euclidean distance (ms) between experiment and simulation is plotted here. Right panel (B) corresponds to zooming in on the red square on the left (A). Point B is the best fit (Euclidean distance = 0.11), it corresponds to the pair S_SS_=5.7% and M_SS_=8.6%, i.e. proliferation rate is 21.0 % (/day), G0/G1 phase is 97.7 hours and G2/M phase is 9.8 hours. (In our fitting procedure, we use a resolution of 0.01% so that the best fit is actually S_SS_=5.74% and M_SS_=8.55% with Euclidean distance of 0.01, but for graphical purposes we do not use this resolution in the example). In order to quantify the variability of the fit according to parameters, we take all fits with a maximum Euclidean distance with experience of 1. These are contained inside a zone centered by point B and bordered by points A and C. In this case, S_SS_ varies between 5.4% and 6.1% (red double arrow), i.e. between proliferation rates of 19.9% and 22.5%. On the other hand, M_SS_ varies between 7.3 and 9.8% (yellow double arrow). Thus G0/G1 phase varies between 93.9 and 100.1 hours, and G2/M phase between 7.9 and 11.6 hours, leading to an inter-mitotic time varying from 4.5 to 4.9 days in this mouse. "Confidence intervals" for our fitting estimates of proliferation rates correspond to the variability of proliferation rates with a distance to experiment of less than 1 (red double arrow).

For better visibility we choose the free parameters to be (S+S')_SS_ and (M+M')_SS_, which we will call S_SS_ and M_SS_ for convenience, i.e. proportions of cells in S phase and G2/M phase at steady-state (Hyp.4), since S_SS_ is directly proportional to proliferation rate. In this case, proliferation rate is equal to p=a_M_M_SS_=a_S_S_SS_=S_SS_/6.5 (Hyp.7). This choice of parameters is arbitrary and has no incidence on results, since all results are fixed by two free parameters. Mean duration of G0/G1 phase is 1/a_G_ with a_G_=a_S_S_SS_/G_SS_=a_S_S_SS_/(100-S_SS_-M_SS_), and mean duration of G2/M phase is 1/a_M_ with a_M_=a_S_S_SS_/M_SS_. After having run 160,000 simulations (S_SS_ and M_SS_ each ranging from 0.1% to 40% every 0.1%), the best fit is the pair of parameters, which minimizes the Euclidean distance between experiment and simulation. In this example, Euclidean distance between experiment and simulation is represented in the figure below.

To take into account the variability of parameters giving a good fit, we define "confidence intervals" for our fitting estimates of proliferation rates, corresponding to the variability of proliferation rates with a distance to experiment of less than 1. This limit of 1 is based on the fact that our flow cytometry data (and also our fitting grid) is precise to the hundredth of a percent (0.01%), and we therefore judge it appropriate to define 1 as a limit distance between experiment and simulation for calculating our confidence intervals. It is however of course arbitrary, and the size of confidence intervals are indeed directly related to the value of this limit. We provide confidence intervals for proliferation rates of all sixteen mice in whole thymus and whole spleen in **S6 Table**.

**Calculation of standard deviations for estimates of proliferation rates.**

To compute standard deviations for our estimated parameters, we use the Hessian matrix defined as $\nabla^{2}f(x^{*})$ where $x^{*}$ is the point in parameter space which corresponds to the best fit, i.e. $(S_{SS}^{*}{,M}_{SS}^{*})$.

We compute the Hessian matrix in the following way:

$$\nabla^{2}f\left( x^{*} \right)={J\left( x^{*} \right)}^{T}J\left( x^{*} \right)+Q(x^{*})$$

where we use the Jacobian matrix :

$$J\left( x^{*} \right)=\left( \begin{matrix} \frac{\partial r_{G}(x^{*})}{\partial S_{SS}} & \frac{\partial r_{G}(x^{*})}{\partial M_{SS}} \\ \frac{\partial r_{M}(x^{*})}{\partial S_{SS}} & \frac{\partial r_{M}(x^{*})}{\partial M_{SS}} \end{matrix} \right)$$

with $r_{G}(x^{*})=G_{sim}(x^{*})-G_{exp}$ and $r_{M}\left( x^{*} \right)=M_{sim}\left( x^{*} \right)-M_{exp}$ (and we neglect the term $Q(x^{*})$ with second-order derivatives which approximates to zero close to $x^{*}$).

We compute numerically the Jacobian in the following way:

$$J\left( x^{*} \right)=\left( \begin{matrix} \frac{r_{G}\left( S_{SS}^{*}+\delta{,M}_{SS}^{*} \right)-r_{G}\left( S_{SS}^{*}{,M}_{SS}^{*} \right)}{\delta} & \frac{r_{G}\left( S_{SS}^{*}{,M}_{SS}^{*}+\delta\right)-r_{G}\left( S_{SS}^{*}{,M}_{SS}^{*} \right)}{\delta} \\ \frac{r_{M}\left( S_{SS}^{*}+\delta{,M}_{SS}^{*} \right)-r_{M}\left( S_{SS}^{*}{,M}_{SS}^{*} \right)}{\delta} & \frac{r_{M}\left( S_{SS}^{*}{,M}_{SS}^{*}+\delta\right)-r_{M}\left( S_{SS}^{*}{,M}_{SS}^{*} \right)}{\delta} \end{matrix} \right)$$

with $\delta=0.1$

We can then calculate the variance-covariance matrix D:

$$D=\sigma^{2}{(\nabla^{2}f(x^{*}))}^{-1}$$

with $\sigma^{2}={r_{G}\left( x^{*} \right)}^{2}+{r_{M}(x^{*})}^{2}$

The diagonal elements of *D* correspond to the variances of the parameters, so finally we can compute standard deviations for each parameter:

$\sigma_{S_{SS}^{*}}=\sqrt{D_{11}}$ and $\sigma_{M_{SS}^{*}}=\sqrt{D_{22}}$

The standard deviation for the proliferation rate is equal to $a_{S}\sigma_{S_{SS}^{*}}$, since $p=a_{S}S_{SS}$.

We present standard deviations for our proliferation rate estimates in **S6 Table**.
